# Anomalous Dynamics of Superparamagnetic Colloidal Microrobots with Tailored Statistics

**DOI:** 10.1002/smll.202506538

**Published:** 2025-10-28

**Authors:** Alessia Gentili, Rainer Klages, Giorgio Volpe

**Affiliations:** ^1^ Department of Chemistry University College London 20 Gordon Street London WC1H 0AJ UK; ^2^ Centre for Complex Systems School of Mathematical Sciences Queen Mary University of London London E1 4NS UK; ^3^ London Mathematical Laboratory London W6 8RH UK

**Keywords:** active colloids, anomalous diffusion, fractional Brownian motion, Lévy walks, microrobots, programmable navigation, stochastic dynamics

## Abstract

Living organisms have developed advanced motion strategies for efficient space exploration, serving as inspiration for the movements of microrobots. These real‐life strategies often involve anomalous dynamics displaying random movement patterns that deviate from Brownian motion. Despite their biological inspiration, autonomous stochastic navigation strategies of current microrobots remain much less versatile than those of their living counterparts. Supported by theoretical reasoning, this work demonstrates superparamagnetic colloidal microrobots with fully customizable stochastic dynamics displaying the entire spectrum of anomalous diffusion, from subdiffusion to superdiffusion, across statistically significant spatial and temporal scales (covering at least two decades). By simultaneously tuning microrobots' step‐length distribution and, critically, their velocity autocorrelation function with magnetic fields, fundamental anomalous dynamics are reproduced with tailored properties mimicking Lévy walks and fractional Brownian motion. These findings pave the way for programmable microrobotic systems that replicate optimal stochastic navigation strategies found in nature for applications in medical robotics and environmental remediation.

## Graphical abstract

Superparamagnetic microrobots are demonstrated to navigate with advanced stochastic motion strategies, producing the entire spectrum of anomalous diffusion, from subdiffusion to superdiffusion, over at least two decades in space and time.

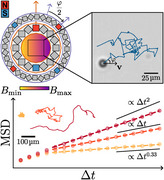

## 1 Introduction

Living organisms have evolved efficient locomotion strategies to navigate complex landscapes, search their surroundings, and improve their fitness.^[^ ^1^, ^2^ ^]^ Selecting an optimal navigation strategy maximizes their ability to locate resources, reach targets, and evade threats.^[^ ^1^, ^2^ ^]^ Often, optimal strategies yield deviations from normal diffusion known as anomalous diffusion.^[^ ^3^ ^]^ These processes are characterized by a non‐linear power‐law scaling of the mean squared displacement (MSD) in time, MSD(*t*) ∼ *t* ^μ^, where *μ* is the anomalous diffusion exponent, including superdiffusion (*μ* > 1) and subdiffusion (*μ* < 1), as opposed to normal diffusion (*μ* = 1).^[^ ^4^ ^]^ Popular stochastic models describing anomalous dynamics in random navigation problems are (superdiffusive) Lévy walks, featuring heavy‐tailed step‐length distributions,^[^ ^5^, ^6^ ^]^ and fractional Brownian motion, showing long‐range correlations with both superdiffusion and subdiffusion.^[^ ^7^, ^8^ ^]^

Inspired by these biological scenarios,^[^ ^9^ ^]^ self‐propelled nano‐ and microrobots have been designed for targeted applications in, e.g., nanomedicine^[^ ^10^ ^]^ and environmental remediation.^[^ ^11^ ^]^ Among these engineered systems, active colloids are widely recognized as synthetic models for living matter,^[^ ^12^, ^13^ ^]^ with significant potential for microrobotic applications due to their simplicity, versatility and ease of fabrication.^[^ ^14^ ^]^

Although advanced autonomous stochastic navigation strategies displaying anomalous dynamics have been successfully implemented and validated in macroscale robotics,^[^ ^15^, ^16^ ^]^ hardware miniaturization constraints have posed significant hurdles to implement the same on smaller scales. Beyond numerical studies,^[^ ^6^, ^17^, ^18^, ^19^, ^20^, ^21^ ^]^ enhanced diffusion (*μ* = 1), and directed motion (*μ* → 2) continue to be the dominant types of fully autonomous navigation mechanisms for active colloids.^[^ ^22^, ^23^, ^24^ ^]^ Attempts at more advanced navigation strategies require information to be stored in the environment^[^ ^25^, ^26^ ^]^ or external feedback loops to correct and steer trajectories in real time,^[^ ^27^, ^28^, ^29^, ^30^, ^31^, ^32^, ^33^ ^]^ for example to implement reinforcement learning‐based approaches.^[^ ^34^, ^35^, ^36^, ^37^ ^]^ However, autonomous stochastic strategies resembling anomalous diffusion patterns as in living organisms remain elusive for active colloids, where, only on rare occasions, short trajectories compatible with Lévy walks have been observed with limited statistics and without robust control over long‐term dynamics or precise control of the anomalous diffusion exponent.^[^ ^38^, ^39^ ^]^

Here, unlike previous experiments, we demonstrate superparamagnetic colloidal microrobots driven by external magnetic fields that move according to fundamental anomalous diffusion patterns with fully tailored statistics spanning the entire spectrum of anomalous diffusion, from subdiffusion (*μ* < 1) to superdiffusion (*μ* > 1), and over statistically significant temporal and spatial scales (covering at least two decades). Supported by theoretical reasoning, we achieve fine control over the microrobots' long‐term dynamics by simultaneously tuning their step‐length distribution and, critically, their velocity autocorrelation function. Thanks to this fine control, our microrobots describe 2D trajectories displaying anomalous dynamics compatible with Lévy walks and fractional Brownian motion with tailored anomalous diffusion exponents, hence better mimicking natural stochastic navigation patterns.^[^ ^1^, ^2^ ^]^

## 2 Results

### 2.1 Anomalous Dynamics of Colloidal Microrobots in the Comoving Frame

**Figure 1:**
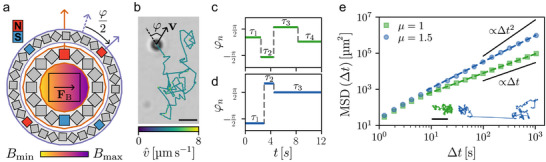
Anomalous diffusion of superparamagnetic colloidal microrobots in the comoving frame. a) Two concentric Halbach cylinders (inner dipole; outer quadrupole) generate a linear magnetic field **B** (color gradient) and a constant force **F**_B_ (black arrow) on a sample of colloidal microrobots (black rectangle). The cylinders' magnetic north (N, red) and south (S, blue) are shown. Rotating the quadrupole (purple arrows) around the fixed dipole (orange arrow) reorients **F**_B_ and the microrobots' direction. Two consecutive rotations define their turning angle *φ* as twice the quadrupole rotation angle. b) Trajectory of a colloidal microrobot moving at approximately constant speed (4.6 ± 0.8 µm $s^{-1}$) in a comoving frame defined by its velocity vector **v** and *φ*. Scale bar: 25 µm. c–d) Bespoke sequences *φ*_*n*_ in time *t* of uniformly distributed *φ* in [−π, π), obtained by sampling the quadrupole rotation time *τ*_*n*_ (solid lines) from c) a half‐Gaussian and d) a power‐law (anomalous exponent *μ* = 1.5) distribution. e) Time‐averaged mean squared displacements (MSD, symbols) and trajectories (inset) of individual microrobots yielding long‐time normal diffusion (*μ* = 1) and superdiffusion (*μ* = 1.5) as confirmed by a logarithmic curve fit (dashed lines). Diffusive (∝ Δ*t*) and ballistic (∝ Δ*t* ^2^) slopes shown for reference. Scale bar: 1 mm.

In **Figure** **1**, we show typical trajectories of microrobots yielding anomalous dynamics. Our microrobots are colloidal superparamagnetic silica spheres of diameter 13.8 ± 0.4 µm driven by external planar rotating magnetic fields (Experimental Section). We use two concentric Halbach cylinders,^[^ ^40^ ^]^ a dipole and a quadrupole, to generate a constant magnetic field gradient |∇|**B**|| ≈ 0.9  T  m^−1^ (Figure 1a; Figure S1 and Table S1 (Supporting Information) and Experimental Section). This gradient translates into a constant magnetic force $F_{B}=\left|m|\nabla |B\right|$, where **m** is the particles' magnetic moment, that drives the colloidal microrobots at constant speed in its direction, $v_{c}\;=\;\left|v\right|\;=\;\frac{|F_{B}|}{6\pi \eta R}$ (Experimental Section), where **v** is the particle's velocity, *R* its radius, and *η* the fluid's viscosity.^[^ ^41^ ^]^ By rotating the quadrupole around the fixed dipole at discrete times *t*_*n*_ with *n* an integer (Figure 1a, Experimental Section), we can reorient **F**_B_ and, hence, the microrobots' motion direction to generate extended tailored trajectories in a 2D comoving frame (Figure 1b). This is a coordinate frame that defines the microrobot's motion in terms of its speed |**v**| ⩾ 0 and turning angle *φ* ∈ [−π, π), where here only the latter changes at times *t*_*n*_ (Figure 1c,d, Supporting Text). Originally introduced by Ross and Pearson about a century ago (Supporting Information), this coordinate frame has been used to analyze and model foraging organisms in movement ecology^[^ ^42^ ^]^ and to formulate advanced stochastic processes such as 2D Lévy walks.^[^ ^6^, ^43^ ^]^ Arguably, the comoving frame is also the most natural one to study the (anomalous) dynamics of active agents, such as living organisms and robots, driven by an internal source of randomness generated by the agents themselves^[^ ^16^, ^44^ ^]^ or to implement dynamics from the agents' perspective using external fields as in this work. Generating stochastic dynamics in a comoving frame implies a fundamental change of perspective compared to defining stochastic processes in a more standard fixed Cartesian frame (Supporting Information). Assuming overdamped dynamics, two decoupled time‐discrete stochastic equations allow us to formulate our microrobot's 2D motion in the comoving frame as (Supporting Information)^[^ ^44^ ^]^

(1) $$\begin{array}{ccc} \varphi_{n} & = & \xi_{\varphi ,n} \end{array}$$

(2) $$\begin{array}{ccc} v_{n} & = & \xi_{v,n} \end{array}$$

where *ξ*_φ,*n*_ and *ξ*_*v*,*n*_ represent arbitrarily complex noise terms driving each coordinate's dynamics sampled at (non‐necessarily equally spaced) discrete times *t*_*n*_ = *t*_*n* − 1_ + *τ*_*n*_ with *τ*_*n*_ the quadrupole rotation time (Experimental Section, Figure 1). Given constant speed, the microrobot runs a distance *ℓ*_*n*_ = *v*_c_τ_*n*_ ballistically during flight time *τ*_*n*_. If we sample *τ*_*n*_ from an arbitrary noise distribution *ξ*_τ,*n*_, we can replace Equation (2) with (Supporting Information)

(3) $$\begin{array}{ccc} \ell_{n} & = & v_{c}\xi_{\tau ,n} \end{array}$$

where the step length and flight time distributions are related by *ξ*_ℓ,*n*_ = *v*_c_ξ_τ,*n*_. By defining appropriate distributions for *ξ*_φ,*n*_ (through the quadrupole rotation angle) and *ξ*_τ,*n*_ (through the quadrupole rotation time) and by sampling independent and identically distributed random variables from these distributions in time, random walks with different statistical properties can be generated in the comoving frame experimentally under the constraint of constant speed (Figure 1, Experimental Section, Supporting Information). For example, sampling *ξ*_φ,*n*_ from the uniform distribution on the circle, our microrobots can describe trajectories yielding long‐time normal diffusion or superdiffusion (Figure 1e; Movie S1, Supporting Information) when *ξ*_τ,*n*_ is sampled from either a half‐Gaussian distribution^[^ ^45^ ^]^ (Figure 1c) or a power‐law distribution with exponent *α* = 3 − *μ* as in the uniform model of 2D Lévy walks^[^ ^43^ ^]^ (Figure 1d for *α* = *μ* = 1.5) (Experimental Section, Supporting Information). Unlike the normal diffusion case, sampling *τ*_*n*_ from a heavy‐tailed power‐law distribution with exponent *μ* leads to occasional long‐lasting flight times during which the microrobot moves ballistically in the direction of the externally applied magnetic field before a reorientation event occurs due to a random field rotation. Under our experimental constraint of constant speed, these rare long‐lasting flight times translate into occasional large spatial displacements according to *ℓ*_*n*_ = *v*_c_τ_*n*_ that are typical of a Lévy walk with exponent *α* = 3 − *μ* and contribute disproportionally to the MSD, making it increase faster than linear, i.e. superdiffusively (Figure 1e).^[^ ^1^, ^5^ ^]^ The superdiffusive trajectory in Figure 1e indeed shows that the microrobot performs occasional long jumps displaying the typical spatial features of Lévy walks (Movie S1, Supporting Information). The random nature of these long jumps is confirmed by the mean displacement of the microrobot's trajectory being nearly zero (the absolute value of the mean displacement over the whole trajectory is 1.16 µm < 0.17*R*, with *R* the particle's radius). The narrow distributions of the instantaneous speed $\hat{v}$ for each trajectory confirm that our microrobots move at an approximately constant speed (Figure S2, Supporting Information), validating the use of Equations (1) and (3) to formulate their motion. Since the microrobot's reorientation timescale is controlled by the implemented stochastic dynamics rather than rotational Brownian motion, our colloidal microrobots have a controllable variable average step length (from 12 µm for normal diffusion to 16 µm for superdiffusion) even at constant speed, unlike systems governed by enhanced diffusion.^[^ ^22^ ^]^ A long‐time fit of the time‐averaged MSD calculated from each trajectory (for Δ*t* > 8 s, i.e., above the short‐time persistence of the trajectory due to the magnetic drive)^[^ ^46^ ^]^ confirms the two desired diffusion regimes over two decades (Figure 1e, Experimental Section). From the fits, we indeed estimate the anomalous diffusion exponents to be $\hat{\mu }=1.0653\pm 0.0002$ and $\hat{\mu }=1.5473\pm 0.0004$, respectively. Our microrobots therefore travel distances two orders of magnitude longer than their own size while reliably maintaining the desired anomalous diffusion dynamics (Experimental Section).

### 2.2 Analysis of Microrobots' Trajectory Statistics

**Figure 2:**
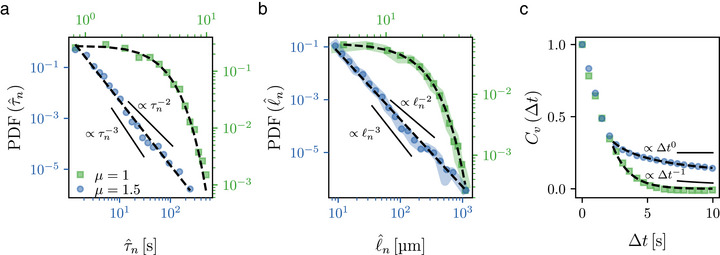
Tailored diffusion statistics of superparamagnetic colloidal microrobots. a,b) Probability distribution functions (PDF) of a) flight times ${\hat{\tau }}_{n}$ between turns and b) step lengths ${\hat{\ell }}_{n}$ extracted from three individual microrobots' trajectories for the cases of normal diffusion (*μ* = 1, green squares) and superdiffusion (*μ* = 1.5, blue circles) as in Figure 1e. c) Respective normalized time‐averaged velocity autocorrelation *C*_*v*_ as a function of lag time Δ*t* for the individual microrobots' trajectories in Figure 1e . In (a–c), fitting curves to the functions (dashed lines) show exponential and power‐law scaling respectively consistent with normal diffusion (*μ* = 1) and superdiffusion (*μ* = 1.5, Table S2, Supporting Information). The thick background lines in (b) represent $\mathrm{PDF}(\left\langle \hat{v}\right\rangle {\hat{\tau }}_{n})$, showing that $\mathrm{PDF}\left({\hat{\ell }}_{n}\right)∼\mathrm{PDF}\left(\left\langle \hat{v}\right\rangle {\hat{\tau }}_{n}\right)$ with $\left\langle \hat{v}\right\rangle$ the microrobot's measured mean instantaneous speed (Figure S2, Supporting Information). The axis colors in (a,b) reflect those of the respective distributions. Diffusive (a: $\propto \tau_{n}^{-3}$; b: $\propto \ell_{n}^{-3}$; c: ∝ Δ*t* ^−1^) and ballistic (a: $\propto \tau_{n}^{-2}$; b: $\propto \ell_{n}^{-2}$; c: ∝ Δ*t* ^0^) limits shown for reference.

A deeper analysis of the experimental trajectories' statistics, based on their segmentation with the detected turning points (Figure S3, Supporting Information, Experimental Section), further confirms that the final microrobots' dynamics are consistent with the desired diffusion regimes (**Figure** **2**). Beyond the mean squared displacements (Figure 1e), we can extract the flight times ${\hat{\tau }}_{n}$, the step lengths ${\hat{\ell }}_{n}$ and the turning angles ${\hat{\varphi }}_{n}$ of our microrobots directly from each trajectory (Experimental Section). The step lengths ${\hat{\ell }}_{n}$ depend linearly on the respective flight times ${\hat{\tau }}_{n}$ (Figure S4, Supporting Information), thus providing an independent confirmation of the microrobots' approximately constant speed. The probability distribution functions (PDFs) of ${\hat{\varphi }}_{n}$, ${\hat{\tau }}_{n}$ and ${\hat{\ell }}_{n}$ (Figure 2a,b and Figure S5, Supporting Information) confirm that the microrobots are reproducing the desired distributions as defined by the quadrupole's rotation angle and time (Experimental Section): for both trajectories in Figure 1e, the distribution of the turning angle is uniform on the circle (Figure S5a,b, Supporting Information), thus matching the intended sampling specified by the quadrupole's rotation in line with Equation (1) (i.e., $\mathrm{PDF}\left({\hat{\varphi }}_{n}\right)∼\mathrm{PDF}\left(\varphi_{n}\right)$, Supporting Information); both distributions of ${\hat{\ell }}_{n}$ and ${\hat{\tau }}_{n}$ show exponential and power‐law scaling (with $\hat{\mu }\approx 1.5$ over two decades, Table S2, Supporting Information), respectively consistent with normal diffusion (*μ* = 1) and superdiffusion for *μ* = 1.5 (Figure 2a,b; Figure S5c, Supporting Information)^[^ ^43^ ^]^, thus matching the intended sampling specified by the quadrupole's rotation times in line with Equation (3) (i.e., $\mathrm{PDF}\left({\hat{\ell }}_{n}\right)∼\mathrm{PDF}\left(\left\langle \hat{v}\right\rangle {\hat{\tau }}_{n}\right)∼\mathrm{PDF}\left(\left\langle \hat{v}\right\rangle \tau_{n}\right)$, Supporting Information). The time‐averaged experimental velocity auto‐correlation function *C*_*v*_(Δ*t*) at lag times Δ*t* is also in agreement with theoretical expectations for the two regimes (Figure 2c, Experimental Section).^[^ ^23^, ^47^ ^]^ The tail of this function decays as an exponential for the diffusive case due to the short‐term persistence in the magnetic field^[^ ^23^ ^]^ and, asymptotically, as a power‐law ($∼\Delta t^{\hat{\mu }-2}$ with $\hat{\mu }=1.434\pm 0.005$, Table S2, Supporting Information) for the superdiffusive case, as expected for unbiased Lévy walks.^[^ ^5^ ^]^

### 2.3 Tuning Microrobots' Step‐Length Distributions

**Figure 3:**
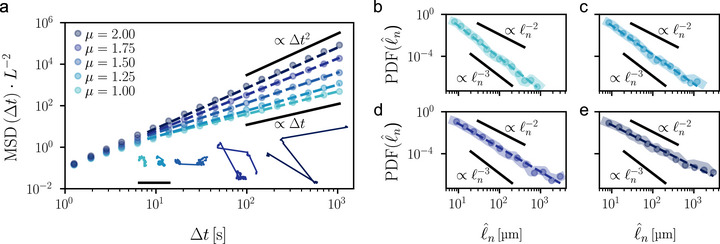
Tailoring microrobots' superdiffusion by controlling step‐length distributions. a) Normalized time‐averaged mean squared displacements (MSD, dots) yielding long‐time superdiffusion for individual microrobots' trajectories (inset) generated according to Equations (1) and (3) by sampling the turning angle *φ*_*n*_ from the uniform distribution on the circle and the flight time *τ*_*n*_ from power‐law distributions of varying exponent *α* = 3 − *μ* between the normal diffusive (*μ* = 1) and ballistic (*μ* = 2) limits. Case for *μ* = 1.5 as in Figure 1e. Fit lines (dashed lines) confirm the different superdiffusive regimes (Table S2, Supporting Information). The MSDs are normalized to the square of each microrobot's short‐term drift distance *L* in the driving magnetic field. Scale bar: 5 mm. b–e) Probability distribution functions (PDF, dots) of experimental step lengths ${\hat{\ell }}_{n}$ from three different microrobot's trajectories each for b) *μ* = 1, c) μ = 1.25, d) *μ* = 1.75, and e) *μ* = 2. $\mathrm{PDF}\left({\hat{\ell }}_{n}\right)∼\mathrm{PDF}\left(\left\langle \hat{v}\right\rangle \;{\hat{\tau }}_{n}\right)$ (thick background lines). Case for *μ* = 1.5 in Figure 2b. Fit lines (dashed lines) show power‐law scalings ($∼{\hat{\ell }}_{n}^{\hat{\mu }-4}$) consistent with the desired ground‐truth values of *μ* (Table S2). Diffusive (a: ∝ Δ*t*; b‐e: $\propto \ell_{n}^{-3}$) and ballistic (a: ∝ Δ*t* ^2^; b‐e: $\propto \ell_{n}^{-2}$) limits shown for reference.

The statistics in Figures 1 and 2 demonstrate that our colloidal microrobots can perform superdiffusion consistent with the uniform model of 2D Lévy walks over two decades in space and time.^[^ ^43^ ^]^ For our approach to be truly versatile, control over the anomalous diffusion exponent *μ* is desirable, since this parameter allows us to tune the average step length of our microrobots even at constant speed. **Figure** **3** shows the possibility of tuning the values of *μ* between the diffusive (*μ* = 1) and ballistic (*μ* = 2) limits by controlling the distributions of the step lengths *ℓ*_*n*_. By sampling *φ*_*n*_ from the uniform distribution on the circle and *τ*_*n*_ from power‐law distributions of varying exponent *α* = 3 − *μ* (Figure S6, Supporting Information),^[^ ^43^ ^]^ our microrobots can describe trajectories in the comoving frame according to Equations (1) and (3) yielding different regimes of superdiffusion in a controllable way under the experimental constraint of constant speed (Table S2, Supporting Information). Figure 3a (inset) shows example trajectories for different values of *μ*: as the anomalous diffusion exponent increases, the microrobots tend to move ballistically over longer distances before a random change in orientation occurs (Movie S2, Supporting Information). Importantly, the four independent measurements $\hat{\mu }$ of the anomalous diffusion exponent obtained from fitting MSDs (Figure 3a, $∼\;\;\Delta t^{\hat{\mu }}$), probability distribution functions of flight times ${\hat{\tau }}_{n}$ (Figure S6, Supporting Information $∼\;{\hat{\tau }}_{n}^{\hat{\mu }-4}$), probability distribution functions of step lengths ${\hat{\ell }}_{n}$ (Figure 3b–e, $∼\;{\hat{\ell }}_{n}^{\hat{\mu }-4}$) and velocity autocorrelation functions (Figure S7, Supporting Information $∼\;\;\Delta t^{\hat{\mu }-2}$) all scale in agreement with theoretical expectations for Lévy walks at the respective ground‐truth value of *μ* (Table S2, Supporting Information).^[^ ^5^ ^]^ Consistent with this scaling, the mean step length of the microrobots also increases with increasing *μ*, from ≈ 11 µm at *μ* = 1 to ≈ 118 µm at *μ* = 2. The ability to precisely tune the anomalous diffusion exponent is particularly beneficial, as different environments may require distinct optimal search strategies. For example, Lévy walks have been shown to be highly efficient in search problems,^[^ ^1^ ^]^ but their optimality can depend on the specific value of *μ* according to environmental characteristics.^[^ ^6^, ^48^ ^]^ For example, while trajectories with *μ* = 2 can be advantageous in terms of search efficiency in homogeneous environments, complex or obstructed landscapes can shift the optimum toward intermediate anomalous diffusion exponents.^[^ ^6^ ^]^ Consistently with this notion, when performing Lévy walks on a complex surface with micro‐obstacles (≈ 20% fractional surface coverage, Figure S8, Supporting Information, Experimental Section), our microrobots with *μ* = 1.50 fare better (by at least 50%) than their diffusive and ballistic counterparts in terms of area exploration rate, as they reduce oversampling compared to microrobots with *μ* = 1 and spend less time stuck at obstacles compared to those with *μ* = 2 (Figure S8, Supporting Information).

### 2.4 Tuning Microrobots' Velocity Autocorrelation Functions

**Figure 4:**
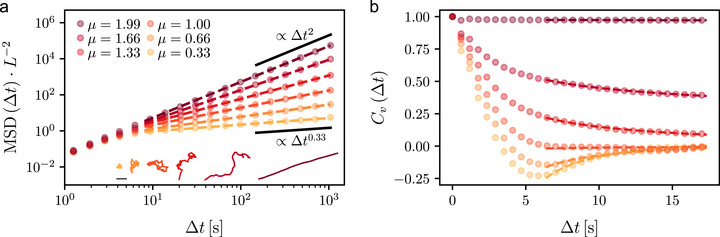
Tailoring anomalous diffusion by controlling the microrobots' velocity autocorrelation function. a) Normalized time‐averaged mean squared displacements (MSD, dots) yielding different anomalous dynamics at long times, from subdiffusion (*μ* < 1) to superdiffusion (*μ* > 1) through normal diffusion (*μ* = 1), for individual microrobots' trajectories (inset) consistent with fractional Brownian motion (Experimental Section, Supporting Information). Fit lines (dashed lines) confirm the different anomalous diffusion regimes (Table S3, Supporting Information). The MSDs are normalized to the square of each microrobot's short‐term drift distance *L* in the driving magnetic field. Subdiffusive (∝ Δ*t* ^0.33^) and ballistic (∝ Δ*t* ^2^) limits shown for reference. Scale bar: 100 µm. b) Respective normalized time‐averaged velocity autocorrelation functions *C*_*v*_ (dots) as a function of lag time Δ*t* calculated from the individual trajectories in (a) for different ground‐truth values of *μ*. Fitting the tail of the data with a quadratic polynomial scaled by a power law (dashed lines) confirms the asymptotic scaling characteristic of fractional Brownian motion at different values of *μ* ($∼\hat{\mu }\left(\hat{\mu }-1\right)\Delta t^{\hat{\mu }-2}$, Table S3, Supporting Information).

The superdiffusive dynamics discussed so far belong to a class of memoryless anomalous dynamics, which means that the microrobot's persistent motion does not depend on its past steps in the trajectory. Introducing memory into diffusive dynamics enables the adoption of both persistent and antipersistent motions, the trade‐off of which can optimize time efficiency versus area coverage in space exploration tasks.^[^ ^2^ ^]^ Such anomalous diffusion dynamics arise when the agent's displacements are not independent but correlated in time. A famous example for this type of dynamics is fractional Brownian motion where the driving noise is no longer white but colored.^[^ ^7^ ^]^ This stochastic process can generate the whole spectrum of anomalous diffusion under parameter variation, from subdiffusion (*μ* < 1) to superdiffusion (*μ* > 1) through normal diffusion (*μ* = 1).^[^ ^7^ ^]^ Experimentally, as there is currently no self‐consistent formulation of fractional Brownian motion in the comoving frame in terms of stochastic equations of motion, we implemented microrobots whose motion satisfies Equations (1) and (3) by pregenerating sequences of flight times *τ*_*n*_ and turning angles *φ*_*n*_ that yield an analogue of 2D fractional Brownian motion in the comoving frame under the constraint of constant speed (Experimental Section, Supporting Information). These preprogrammed sequences of turning angles and flight times are directly derived from fractional Brownian motion trajectories numerically generated in the 2D Cartesian frame (Experimental Section). Following the protocol described in the Supporting Information, we then transformed these preprogrammed dynamics into dynamics in the comoving frame that replicate the long‐range temporal correlations and MSD scaling of fractional Brownian motion. In analogy to the transformation between Lévy flights and walks, we refer to this fractional Brownian motion‐like process as fractional Brownian walks, a constant‐speed analogue that retains fractional Brownian motion's key statistical features. **Figure** **4** shows that, as *μ* increases, the corresponding trajectories (Figure 4a, inset) become less localized and more ballistic (Movie S3, Supporting Information). The microrobots' tendency to turn backward (negative persistence) reduces in favor of its forward propagation (positive persistence) (polar plots, Figure S9, Supporting Information). A long‐time fit of the time‐averaged MSDs calculated from each trajectory (Figure 4a; Table S3, Supporting Information) confirms the shift from subdiffusion (sublinear MSD, *μ* < 1) to superdiffusion (superlinear MSD, *μ* > 1) through normal diffusion (linear MSD, *μ* = 1), when the microrobots' velocities show a transition from negative to positive correlations (Figure 4b; Table S3, Supporting Information) in agreement with theoretical expectations (Experimental Section, Supporting Information).^[^ ^49^ ^]^ Therefore, these two consistent independent measurements (i.e., by fitting the MSD and *C*_*v*_) of the anomalous diffusion exponent associated with each trajectory strongly support the tailored generation of different types of anomalous diffusion dynamics, compatible with fractional Brownian motion, for our colloidal superparamagnetic microrobots by the spatio‐temporal control of their turning angles and flight times in the comoving frame (Table S3, Supporting Information).

## 3 Conclusion

We have demonstrated colloidal superparamagnetic microrobots capable of programmable anomalous dynamics compatible with 2D models of normal diffusion,^[^ ^23^ ^]^ Lévy walks,^[^ ^5^ ^]^ and fractional Brownian motion.^[^ ^7^ ^]^ Supported by theoretical reasoning, we have implemented these anomalous dynamics in a comoving frame, i.e., a frame moving and rotating with the microrobot, directly capturing the motion from the perspective of the active agent itself. We validated these dynamics over statistically relevant temporal and spatial scales by precisely tuning two key experimental parameters (i.e., the microrobot's turning angles and flight times). Our approach enables the computationally efficient motion planning of colloidal microrobots capable of autonomous navigation based on diverse advanced random strategies without requiring onboard circuitry^[^ ^9^ ^]^ or the implementation of any external feedback based on the position and velocity of the microrobots.^[^ ^27^, ^28^, ^29^, ^31^, ^32^, ^33^ ^]^ Such autonomous random navigation strategies can prove beneficial in exploring complex unknown environments, where deterministic strategies may struggle and a stochastic approach may be preferred,^[^ ^2^, ^6^, ^50^, ^51^ ^]^ e.g., for manipulation and navigation tasks in therapeutic applications^[^ ^52^ ^]^ and environmental remediation.^[^ ^53^ ^]^ While we used external fields, our work could serve as a steppingstone toward the development of fully autonomous microrobots moving anomalously by exploiting internal sources of randomness instead, as the comoving frame representation we developed here is independent of the microscopic details of the experimental implementation. In the case of fractional Brownian motion, this would also require a self‐consistent formulation of this process' stochastic equations of motion in the comoving frame to be developed first. Beyond advancing the capabilities for autonomous navigation of microrobots, we also anticipate that our framework will provide a robust experimental platform for validating theoretical and predictive models and analysis methods of both ergodic and non‐ergodic anomalous diffusion dynamics in active matter and beyond,^[^ ^54^, ^55^ ^]^ thus contributing to deepen our general understanding of anomalous diffusion processes across various fields and scales, from the life sciences to macroscopic natural and human processes.^[^ ^56^, ^57^, ^58^, ^59^, ^60^, ^61^ ^]^

## 4 Experimental Section

### Materials

Glass microscopy slides (25 mm × 75 mm × 1 mm, Epredia) and glass coverslips (24 mm × 24 mm × 0.14 mm) for sample preparation were purchased from Thermo Fisher and VWR, respectively. The following chemicals were purchased and used as received: acetone (⩾99.8%, Sigma–Aldrich), ethanol (⩾99.8%, Fisher Scientific), ethylene glycol (Sigma–Aldrich), Tween 20 (Sigma–Aldrich), sodium chloride (NaCl, Sigma–Aldrich). Deionized (DI) water (≥18MΩcm, type II Water) was collected from a Milli‐Q purification system. Aqueous colloidal dispersions (5% w/v) of superparamagnetic and plain silica (SiO_2_) particles were purchased from Microparticles GmbH. Parafilm (Bemis Parafilm M Laboratory Wrapping Film), used as spacer for the sample chamber, was purchased from Fisher Scientific. Two‐part epoxy glue (Gorilla Epoxy) for sealing the samples was purchased from RS Components. The neodymium magnets used to build the Halbach cylinders were purchased from K&J Magnetics, Inc. (B666‐N52) and supermagnete (W‐07‐N). PA 2200 (nylon powder) was used to 3D‐print the encasing of the magnets for the cylinders.

### Colloidal Dispersion

As microrobots, superparamagnetic silica (SiO_2_) colloidal particles with a diameter of 13.8 ± 0.4 µm, an iron oxide content greater than 5 wt. %, and a high density of approximately 1.5 g  cm^−3^, as estimated by the manufacturer were used. Before each experiment, the original batch dispersion was gradually diluted in a 50% ethylene glycol and 50% DI water solution by volume to achieve very low particle concentrations (<10^−5^ w/v%) and avoid interparticle interactions in a magnetic field. The viscosity of this solution was approximately four times that of pure DI water^[^ ^62^ ^]^ to reduce the particles' speed when exposed to the high magnetic fields generated by the Halbach cylinders (Figure 1). Typical Péclet numbers range between 4000 and 6000. Such high values indicate that Brownian motion was negligible and directed motion dominated the short‐term dynamics of the particles.^[^ ^22^ ^]^ To prevent the particles from sticking to the glass slides during experiments, small traces (<0.002 v/v%) of a 10% Tween 20 aqueous solution were added to the final dispersion. By preventing sticking and by increasing viscosity to reduce occurrences of particles exiting the field of view, their dynamics were controlled for durations of up to 9 h.

### Sample Chamber

A volume of 62 µl of the colloidal dispersion was confined within a quasi‐2D chamber assembled from a microscope slide (bottom layer, cut to approximately 25 mm × 28 mm × 1 mm) and a coverslip (top layer) using two strips of melted parafilm as spacers to obtain a thickness of ≈ 20 µm. First, both the glass slide and coverslip were cleaned by sequentially immersing them in Coplin jars containing acetone, ethanol and DI water in an ultrasonic bath for 5, 10, and 15 min, respectively. Blowing the slide and coverslip dry with nitrogen gas removed excess water. For experiments with micro‐obstacles only (Figure S8, Supporting Information), the glass slide was additionally prepared with immobilized microparticle clusters serving as obstacles for the microrobots according to a previously developed protocol.^[^ ^63^ ^]^ A ten µl drop of a 1.5 wt.% aqueous suspension of silica microparticles (diameter 20.00 ± 0.64 µm) containing 0.1 M sodium chloride (NaCl) was deposited at the center of the cleaned glass slide. After 2 min to allow the particles to sediment, the drop was dried by capillarity using filter paper at its edge. The slide was then placed on a hot plate at 60 °C for 1 min to promote long‐term adhesion of the microparticle clusters to the glass surface and to remove residual water.^[^ ^63^ ^]^ Finally, the slide was immersed in a Petri dish filled with deionized water for 5 min to dissolve excess salt, followed by gentle nitrogen drying. This method allowed to obtain an average ≈ 20% fractional surface coverage of the obstacles in the entire field of view. After preparation of the sample chamber's bottom layer, two strips of parafilm (approximately 25 mm × 5 mm each) were placed at opposite edges of the glass slide and let them melt on a hotplate at 60 °C, near the melting point of parafilm. Once the parafilm turned transparent (after about 3 min), the coverslip was placed on top, and slight pressure was applied using the flat tip of a pair of tweezers to close the chamber. After cooling and loading the particles' dispersion, the chamber was sealed with two‐part epoxy glue, and let it cure and rest for at least 20 min before each experiment. Due to the small volume and low concentration of colloids in the dispersion, the experimental chamber contained very few particles (typically less than 3), allowing to select and track individual microrobots in each experiment.

### Magnetic Fields with Halbach Cylinders

The constant magnetic field gradient ∇|**B**| needed to drive our superparamagnetic colloidal microrobots at constant speed *v*_c_ = |**v**| in the sample plane was generated using two concentrical Halbach cylinders (Figure 1a; Figure S1, Supporting Information): an inner dipole (Figure S1a, Supporting Information) surrounded by an outer quadrupole (Figure S1b, Supporting Information).^[^ ^40^ ^]^ These cylinders, constituted by circular arrays of permanent magnets (Figure S1c, Supporting Information), can produce controlled magnetic fields entirely within their core while canceling it on the outside. In this case, the axis of the cylinders is aligned along the direction (*z* in Figure S1, Supporting Information) perpendicular to the sample plane (*xy* in Figure S1, Supporting Information). The inner Halbach cylinder produces a strong homogeneous dipolar magnetic field **B** ^D^ in this plane with constant intensity *B*_0_ along the *y*‐axis (Figure S1a,d–f), maximizing the magnetic moment **m** of the superparamagnetic particles and aligning it along the field lines. The outer cylinder generates a weaker quadrupolar magnetic field **B** ^Q^ consisting of two orthogonal linear components in space (i.e., each with a constant derivative of magnitude *G*) (Figure S1b,g–i, Supporting Information). When the two arrays are coaxially aligned, the resulting field **B**(**r**) at position **r** = (*x*, *y*) is linear in space and given by

(4) $$B\left(r\right)=B^{D}\left(r\right)+B^{Q}\left(r\right)=B_{0}\left[\begin{array}{c} 0\\ 1 \end{array}\right]+G\left[\begin{array}{cc} -\cos2\beta & \sin2\beta \\ \sin2\beta & \cos2\beta \end{array}\right]\left[\begin{array}{c} x\\ y \end{array}\right]$$

where 2*β* is the angle of rotation of the magnetic field gradient induced by a *β* rotation of the quadrupole around the dipole.^[^ ^40^ ^]^ The direction of the gradient can therefore be adjusted by rotating the quadrupole with respect to the dipole (Figure 1a), and the difference Δ*β* between two consecutive rotations of the quadrupole defines the microrobot's turning angle *φ* as *φ* = 2Δ*β* (Figure 1a,b). As a result of combining a strong homogeneous dipolar magnetic field with a weaker constantly graded one, the microrobots move in a well‐defined, spatially independent, and adjustable direction defined only by the component of the gradient parallel to **B** ^D^ (the *y*‐component in Equation (4)).^[^ ^41^ ^]^

A discrete version of the Halbach dipole with radius *r*_c_ = 30.05 mm was implemented using *k* = 16 cubic neodymium magnets (grade N52, remanence *B*_R_ ≈ 1.48 T, relative permeability *μ*_R_ = 1.05, side length *a*_m_ = 9.5 mm) with *B*_0_ given by

(5) $$B_{0}=B_{R}\ln\left(\frac{r_{\mathrm{out}}}{r_{\mathrm{in}}}\right)\frac{1}{\sqrt{\mu_{R}}}\frac{\sin(2\pi /k)}{2\pi /k}\frac{k\;a_{m}}{\pi (r_{\mathrm{out}}^{2}-r_{\mathrm{in}}^{2})}\frac{h(6r_{c}^{2}+h^{2})}{{\left(4r_{c}^{2}+h^{2}\right)}^{3/2}}\approx 85\;\mathrm{mT}$$

where *r*_in_ = 23.3 mm and *r*_out_ = 36.8 mm are the cylinder's inner and outer radii, respectively (Figure S1c, Supporting Information), and *h* = *a*_m_ its height.^[^ ^40^ ^]^ The cylinder radius *r*_c_ can be calculated from these two values as their average *r*_c_ = (*r*_in_ + *r*_out_)/2 and was chosen to be more than double the entire sample's size to prevent edge effects due to field inhomogeneities nearer to the magnets. Moreover, the Halbach dipolar cylinder was implemented as a vertical stack of two identical circular arrays separated by 15.3 mm,^[^ ^64^ ^]^ to reduce field inhomogeneity in the *z*‐direction, thus minimizing any possible vertical magnetic drift of the colloids. Stacking the two arrays also produced an expected increase in field intensity by a factor of 1.351 with respect to the prediction in Equation (5). This increase was confirmed using a Gaussmeter (Lake Shore Cryotronics, Inc., Model 420), measuring an average magnetic field of 111.38 ± 0.66 mT (Figure S1d–f, Supporting Information).

Similarly, a discrete version of the Halbach quadrupole was implemented with a larger radius *r*_c_ = 46.7  mm using *k* = 32 cubic neodymium magnets (grade N42, remanence *B*_R_ ≈ 1.32  T, relative permeability *μ*_R_ = 1.05, side length *a*_m_ = 7  mm) with *G* given by (ref. [40])

(6) $$\begin{array}{ccc} G & = & \frac{2B_{R}}{\sqrt{\mu_{R}}}\left(\frac{1}{r_{\mathrm{in}}}-\frac{1}{r_{\mathrm{out}}}\right)\frac{\sin(3\pi /k)}{3\pi /k}\frac{k\;a_{m}^{2}}{\pi (r_{\mathrm{out}}^{2}-r_{\mathrm{in}}^{2})}\\ & & \frac{h(h^{4}+10h^{2}r_{c}^{2}+30r_{c}^{4})}{{\left(4r_{c}^{2}+h^{2}\right)}^{5/2}}\\ & & \approx \;0.9\;{T\;m}^{-1} \end{array}$$

This value was confirmed calculating the gradient (0.98 ± 0.08  T m^−1^) from the magnetic field intensities measured with the Gaussmeter (Figure S1g–i, Supporting Information).

Table S1 (Supporting Information) summarizes all parameters used to implement both Halbach cylinders. All magnets were held in place side by side by plastic molds (one for the dipole and one for the quadrupole), which were 3D‐printed using Selective Laser Sintering (SLS) technology.

### Experimental Setup

Individual microrobots' trajectories were recorded using a custom‐built inverted microscope. The sample rested in the region of homogeneous dipolar field at the center of the Halbach dipole, which was supported by four metal pillars (Thorlabs). The sample holder was uncoupled from the dipole support to reduce transmission of vibrations due to the rotation of the quadrupole around the dipole. For the same reason, the quadrupole was mounted on a third separate support, vertically centered at the sample level, and connected to a high‐speed motorized rotational stage (Zaber, X‐RSB060AD). To reorient the magnetic field gradient instantaneously with respect to the microrobots' dynamics, the quadrupole was rotated at constant angular speed (60 rad s^−1^). This value was chosen because, during the time required by the quadrupole to complete the largest rotation in the experiments (Δ*β* = ±π/2), a microrobot traveled a distance comparable with the localization error on the determination of its centroid (≈ 0.13 µm), thus with negligible influence on the final trajectory. To reduce vibrations, the quadrupole rotation was ramped up to (down from) its maximum speed with a constant angular acceleration (deceleration) of 60 rad s^−2^. For sample illumination, a monochromatic LED (*λ* = 660 nm, Thorlabs, M660L4) equipped with an adjustable collimation adapter (Thorlabs, SM2F32‐A) was mounted on this last support. To acquire long trajectories, the imaging system was also uncoupled from the part of the setup containing the sample. The imaging system was formed by two lenses projecting the image of the sample with a 4× magnification on a monochrome complementary metal–oxide–semiconductor (CMOS) camera (Thorlabs, DCC1545M). This system was mounted on a computer‐controlled two‐axis motorized translation stage (Thorlabs, PT1/M‐Z8) to allow to recenter the microrobot in the field of view of the camera (1.3 mm × 1.6 mm), thus avoiding that the particle exited it in long linear stretches of its motion. Videos of microrobots were recorded with a frame rate of 11.94 frames per second (the inverse of the sampling time δ*t*) using a custom MATLAB program that triggered the camera acquisition. The same program controlled the sequence of rotations of the quadrupole to implement bespoke patterns of anomalous diffusion and the translation of the imaging system based on the microrobot's position. During relatively long quadrupole rotation times (>15 s), video recording was temporarily interrupted to automatically recenter the microrobot to the field of view every 2 s before recording was resumed. The stage was translated with a constant speed of $1\;\mathrm{mm}\;s^{-1}$, approximately 200 times faster than typical particles' speeds, i.e., almost instantaneously compared to the microrobots' dynamics (Figure S2, Supporting Information). To reduce vibrations, the stage translation was ramped up to (down from) its maximum speed with a constant acceleration (deceleration) of $1\;\mathrm{mm}\;s^{-2}$. Full microrobots' trajectories were reconstructed by stitching together individual trajectories (Figure S10, Supporting Information, see Trajectory stitching below) obtained from sequences of videos corresponding to each experiment using homemade Python scripts based on the trackpy package.^[^ ^65^ ^]^ Like this, trajectories were acquired over centimeter‐long scales over extended periods of time (up to 9 h).

### Trajectory Stitching

Full microrobots' trajectories were reconstructed by stitching together individual trajectories from a sequence of *N* consecutive videos. To facilitate stitching, any two consecutive videos respectively finished and started with an at least 1‐s long portion of the same step length *ℓ*_*n*_ in the trajectory (Figure S10, Supporting Information). These portions were reconnected by translating all the *i* points $r_{i}^{j+1}$ of the trajectory (with *j* and *i* both integers) defined in the coordinate system of the (*j* + 1)^th^ video back to the reference system of the *j* ^th^ video (Figure S10, Supporting Information). The origin of the coordinate system associated to each video was at the center of its field of view. As the microrobot was moving ballistically at the time of its recentering, the additional distance it traveled between recordings (Figure S10) was also accounted. The translation between the reference systems of two consecutive videos is then given by

(7) $$\begin{array}{c} r_{i}^{j}=r_{i}^{j+1}+r_{M}^{j}\approx \left(r_{i}^{j+1}-r_{0}^{j+1}\right)+\tau_{\delta }\left\langle {\hat{v}}_{\ell }\right\rangle u_{v}+r_{M}^{j} \end{array}$$

where $r_{i}^{j}$ and $r_{M}^{j}$ are respectively the positions of $r_{i}^{j+1}$ and of the last recorded point of the *j* ^th^ video in its reference system, and $r_{0}^{j+1}$ identifies the first particle's position of the (*j* + 1)^th^ video in its reference system. If the microrobot was moving ballistically at approximately constant speed (as in the experiments, Figure S2, Supporting Information), the vector $r_{0}^{j+1}\approx \tau_{\delta }\left\langle {\hat{v}}_{\ell }\right\rangle u_{v}$, where τ_δ_ was the time elapsed between recordings, $\left\langle {\hat{v}}_{\ell }\right\rangle$ was the average particle's speed in the two recorded portions of the step length being reconstructed, and **u**_*v*_ = (cos(θ), sin(θ)) was the unitary vector in the direction of motion, i.e., also defined by the same step length of the full trajectory which was being reconstructed. Finally, the displacement between the last point of the *j* ^th^ video and the first point of the (*j* + 1)^th^ video was linearly interpolated by resampling with the experimental sampling time δ*t* (the inverse of the frame rate). This procedure was repeated iteratively until the trajectory was fully reconstructed in the coordinate system of the first acquisition video. Importantly, Figure S11 (Supporting Information) verifies that any two reconnected portions in the reconstructed trajectory maintain the same direction of motion (Figure S11b, Supporting Information) and the validity of the relationship $r_{0}^{j+1}\approx \tau_{\delta }\left\langle {\hat{v}}_{\ell }\right\rangle u_{v}$ based on direct measurements of $r_{0}^{j+1}$ from image analysis and measurements of stage displacements (Figure S11b, Supporting Information), thus further confirming our constant speed approximation. The proportionality with slope 1 (Figure S11, Supporting Information) between these two quantities (that is, $r_{0}^{j+1}$ from direct stage displacement measurements and the independent estimate of $\tau_{\delta }\left\langle {\hat{v}}_{\ell }\right\rangle u_{v}$ from the microrobot's constant velocity and the elapsed time between consecutive recordings) enables to rule out the presence of systematic biases and artifacts in the trajectories reconstructed with this stitching protocol.

### Distributions of Quadrupole Rotation Times and Turning Angles

For trajectories yielding normal diffusion (Figure 1 and 2, *μ* = 1), sequences of *N* quadrupole rotation times *τ*_*n*_ were numerically generated from a half‐Gaussian distribution (Figure S5, Supporting Information). The probability density function (PDF) of this distribution is

(8) $$\mathrm{PDF}\left(\tau \right)=\frac{e^{-\tau^{2}/16}}{2\sqrt{\pi }},\;\;\tau \geq 0$$

For trajectories yielding Lévy walks of exponent *α* = 3 − *μ* (Figures 1, 2, 3), sequences of *N* quadrupole rotation times *τ*_*n*_ were numerically generated using the inverse method (Figures S5 and S6, Supporting Information):^[^ ^66^ ^]^ by drawing *r* as a random number from a uniform distribution in [0,1), the variable *τ* = *τ*_min_(1 − *r*)^−1/*α*^ follows a power‐law distribution with exponent (*α* + 1) and lower bound τ_min_

(9) $$\mathrm{PDF}\left(\tau \right)=C\tau^{-(\alpha +1)},\;\;\tau \geq \tau_{\min}$$

where $C=\alpha \tau_{\min}^{\alpha }$ is a normalization constant. A power‐law distribution with a lower bound was preferred over an *α*‐stable Lévy distribution to optimize experimental time by focusing directly on the tail of the distributions. For this purpose, *τ*_min_ = 1 s was set to facilitate the detection of turning points in the trajectories (see Turning point detection).

Finally, sequences of *N* turning angles *φ*_*n*_ were drawn from a uniform distribution over the half‐open interval [−π, π) for both normal diffusion and Lévy walks (Figures S5 and S6, Supporting Information). To simplify the task of detecting turning points, angles were drawn uniformly at random from a discrete set of values space by π/6 in this interval. In all cases, the value of *N* was chosen so that the cumulative sum of all *τ*_*n*_ was at least 3‐h long to observe anomalous diffusion in experiments over at least two decades in space and time.

For fractional Brownian motion, sequences of (*τ*_*n*_, *φ*_*n*_) yielding a constant‐speed analogue of this process in the comoving frame were generated, which satisfies Equation (1) and (3), by adopting the protocol detailed in the Supporting Information. In analogy to the transformation between Lévy flights and walk, realizations of this process were referred as fractional Brownian walks. Briefly, trajectories generated in simulations with a constant flight time ${\tilde{\tau }}_{c}$ and non‐constant Gaussian‐distributed velocities ${\tilde{v}}_{n}$ in a Cartesian frame were transformed into trajectories of the same path topology with constant speed *v*_c_ and non‐constant flight times *τ*_*n*_ in the comoving frame by doing the following (Supporting Information): 2D (time‐discrete) Cartesian fractional Brownian motion was first generated from the Davies‐Harte method^[^ ^67^ ^]^ implemented in the Python package stochastic for all values of the anomalous exponent *μ* used in the experiments; in order to scale the average speed in simulations (${\tilde{v}}_{c}=\left\langle {\tilde{v}}_{n}\right\rangle =\sqrt{\frac{\pi }{2}}\sigma_{\tilde{v}}$ for $\sigma_{\tilde{v}}=1$ µm $s^{-1}$ and ${\tilde{\tau }}_{c}=1\;s$) to a representative a‐priori estimate for the microrobot's experimental average speed *v*_c_ = 4.5 µm $s^{-1}$, Equation (S33) (Supporting Information) was then applied with a scale factor of *κ* = 3.6 (defined by Equation (S23), Supporting Information), thus effectively matching the experimental length scales; finally, the scaled sequences of speeds and turning angles (*v*_*n*_, *φ*_*n*_) associated to each trajectory were transformed into the corresponding (*τ*_*n*_, *φ*_*n*_) sequences for the rotation of the quadrupole implementing the transformation given by Equation (S29) (Supporting Information).

### MSD Calculation and Fitting

For each trajectory, the time‐averaged mean squared displacement (MSD) was calculated at discrete time lags Δ*t* = *m*δ*t* (with δ*t* the experimental sampling time and *m* an integer) as^[^ ^46^ ^]^

(10) $$\mathrm{MSD}\left(\Delta t\right)=\frac{1}{T-\Delta t}\sum_{t=\delta t}^{T-\Delta t}\left[{\left(x(t+\Delta t)-x(t)\right)}^{2}+{\left(y(t+\Delta t)-y(t)\right)}^{2}\right]$$

where **r** = (*x*, *y*) are the trajectory's coordinates sampled at time steps *t* = *p*δ*t* (with *p* an integer), and *T* = *P*δ*t* (with *P* = 12500) is the total number of data points in the MSD calculations. Time‐averaging of the mean squared displacement is appropriate, as normal diffusion and fractional Brownian motion are ergodic processes, and the weak ergodicity breaking of Lévy walks does not affect the power‐law scaling of the MSD in homogeneous environments.^[^ ^54^ ^]^ The value *T* was chosen to be shorter than the trajectory length, but large enough to extract anomalous diffusion exponents by fitting the MSD over at least two decades with strong statistical reliability. The scaling exponent $\hat{\mu }$ of the MSD was estimated with a linear fit in log–log scale in the asymptotic limit (i.e., for Δ*t* > 8 s, after the short‐time persistence transition point, Figures 1, 3, 4). The reported uncertainty associated with the estimated exponent $\hat{\mu }$ (Tables S2 and S3, Supporting Information) corresponds to one standard deviation of the fit parameter.

### Detection of Turning Points

Turning points along microrobots' trajectories were identified based on the detection of local extrema in their velocity. Given that the microrobots move at nearly constant speed (Figure S2, Supporting Information), significant variations of this quantity should primarily reflect directional changes. Experimentally, variations of the absolute value of the acceleration magnitude gradient (|∇|**a**||, Figure S3a, Supporting Information) were used as a noise‐robust empirical proxy to identify these directional changes. To further minimize the impact of the experimental noise, this time series was preprocessed with a Savitzky‐Golay filter with a five‐point kernel,^[^ ^68^ ^]^ implemented with the Python scipy.signal.savgol_filter function. Prominent peaks were then identified using the Python scipy.signal.find_peaks function.^[^ ^69^ ^]^ This method was validated for the independent detection of the turning points directly from the acquired trajectories by comparing their predicted values ${\hat{\tau }}_{n}$ against the ground truth from the sequences *τ*_*n*_ of quadrupole rotations (Figure S3b, Supporting Information). For all trajectories, a F_1_ score of at least 0.83 was achieved. Here, the micro average of the F_1_ score was computed using the Python sklearn.metrics.f1_score function with a tolerance of five data points (≈ 0.42 s),^[^ ^70^ ^]^ i.e., a predicted turning point was considered a true positive if it was within five points of a ground truth value.

### Experimental Distributions of Flight Times and Step Lengths

After identifying the turning points along each trajectory, the probability density functions (PDFs) of the flight times ${\hat{\tau }}_{n}$ and step lengths ${\hat{\ell }}_{n}$ of the particles were calculated (Figures 2 and 3; Figures S5, S6, and S12, Supporting Information). For normal diffusion (Figure 2a,b), the PDFs should decay exponentially in the long‐time limit, which was verified by fitting them to a half‐Gaussian function.^[^ ^3^, ^5^ ^]^ For trajectories yielding Lévy walks (Figures 2a,b and 3b–e), the asymptotic power‐law scaling and corresponding anomalous exponent *μ* were verified with a linear fit of the distribution tails on log–log scale (Table S2, Supporting Information).^[^ ^3^, ^5^ ^]^ To increase tail statistics, data from three different trajectories were combined for each value of *μ*. For fractional Brownian walks, the PDFs should follow a Rayleigh distribution independent of the anomalous diffusion exponent *μ* (Supporting Information), as confirmed experimentally (Figure S12, Supporting Information).

### Experimental Velocity Autocorrelation Functions

For each trajectory, the normalized velocity autocorrelation function (VACF) was calculated as

(11) $$C_{v}\left(\Delta t\right)=\frac{\left\langle v(t)\cdot v(t+\Delta t)\right\rangle }{\left\langle v^{2}\left(t\right)\right\rangle }$$

where **v**(*t*) is the instantaneous microrobot's velocity at time *t*, Δ*t* is the time lag for the calculation of the VACF and 〈…〉 indicates a time average. The microrobot's velocity was calculated as $v\left(t\right)=\frac{r(t+5\delta t)-r(t)}{5\delta }$ with δ*t* the experimental sampling time, i.e., using a time window of 0.42 s (corresponding to five video frames) moving along the trajectory to minimize the impact of the tracking localization noise. For normal diffusion, *C*_*v*_(Δ*t*) follows an exponential decay as expected (Figure 2c).^[^ ^5^, ^23^ ^]^ For trajectories yielding Lévy walks, it was confirmed that *C*_*v*_(Δ*t*) decays as a power law of consistent anomalous diffusion exponent *μ* asymptotically (Figure S7 and Table S2, Supporting Information).^[^ ^5^ ^]^ For fractional Brownian walks, it was confirmed that *C*_*v*_(Δ*t*) decays as the asymptotic functional form characteristic of this process given by $C_{v}\left(\Delta t\right)≃\frac{1}{2}\mu \left(\mu -1\right)\Delta t^{\mu -2}$ for each value of *μ* (Figure 4b; Table S3, Supporting Information).^[^ ^71^ ^]^

### Navigation in Presence of Micro‐Obstacles

Microrobots performing Lévy walks on a surface with micro‐obstacles (Figure S8, Supporting Information) were controlled and recorded as previously described without interrupting the recording to recenter the microrobot within the field of view, as the interaction with the obstacles meant that the microrobot did not leave the field of view within the experimental time. The turning times and angles implemented followed the same distributions as the trajectories in Figure 3a. For each value of *μ*, four different trajectories were recorded, each starting in a different field of view of same fractional surface coverage (≈ 20%). Within the same dataset (i.e., a complete set of trajectories with all values of *μ*), the microrobot was always restarted from approximately the same location for fairer comparison of exploration efficiency. Trajectories were extracted from video recordings using custom Python scripts based on the Trackpy package, after applying a binary mask to remove obstacles from the video frames for ease of tracking. The binary mask was generated from the first frame of each experiment using Otsu's thresholding method (implemented in scikit‐image library) to identify obstacle regions. The microrobot was excluded from the final mask by detecting its position with the Trackpy package and removing a circular region centered on its coordinates. Efficiency was quantified as the average area exploration rate 〈*A*_*t*_〉 extracted from each trajectory. The coordinates were discretized on a square grid with a bin size equal to the diameter of the microrobot (13.81 µm) to calculate the histrogram of the sites it visited. For each trajectory, this 2D histogram was converted into a binary map of uniquely visited sites, where each bin was marked as visited if occupied at least once by the particle. *A*_*t*_ was then defined as the sum of the areas of these uniquely visited bins normalized by the duration of the trajectory.

### Statistical Analysis

All data were processed and analyzed using custom Python scripts. Statistical analyses were limited to descriptive model fitting. Unless differently specified, results were typically presented as fit parameters ± one standard deviation, obtained from the diagonal elements of the fit covariance matrix. All fittings were performed using unweighted least squares (scipy.optimize.curve_fit). Unless otherwise stated, all analyses were performed on a single long trajectory. For all processes, individual trajectories were obtained from video data using the Trackpy library and correspond to single microrobots tracked for at least 1.5 h and up to 9 h with a sampling time of 0.08 s. Trajectory lengths ensured a time range spanning at least two orders of magnitude in the time‐averaged MSD. MSDs were computed as a function of the time lag, and the estimated anomalous diffusion exponent $\hat{\mu }$ was extracted from a linear fit in log–log scale to the asymptotic regime, with the fit slope used to determine $\hat{\mu }$. Probability density functions (PDFs) of flight times (${\hat{\tau }}_{n}$) and step lengths (${\hat{\ell }}_{n}$) were obtained from single trajectories after turning point detection. Turning points corresponded to significant peaks in the absolute gradient of the acceleration magnitude, identified using SciPy's find_peaks function after smoothing with a five‐point Savitzky–Golay filter. PDFs were computed with a custom function that produces normalized histograms using logarithmic bins for Lévy walks and linear bins for fractional Brownian walks and normal diffusion. For Lévy walks, step‐length data from three independent trajectories (three different experiments) per values of *μ* were pooled to improve tail statistics, with the number of step lengths ranging from 838 at *μ* = 2.00 to 40985 at *μ* = 1.00. The tails of the distributions were fitted in log–log scale using unweighted linear regression over at least two decades in step length. For fractional Brownian walks, flight‐time PDFs were fitted in linear scale to a Rayleigh distribution (Equation (S30), Supporting Information) using unweighted least squares. For normal diffusion, both flight‐time and step‐length PDFs were fitted in linear scale to an exponential model using unweighted least squares. The normalized velocity autocorrelation function (VACF) was computed from instantaneous velocities estimated over a five‐frame (0.42 s) window (Equation (11)). VACF decay was fitted to model‐specific forms – exponential for normal diffusion, power law for Lévy walks, and the asymptotic form characteristic of fractional Brownian motion for fractional Brownian walks.

## Conflict of Interest

The authors declare no conflict of interest.

## Author Contributions

Author contributions are defined based on the CRediT (Contributor Roles Taxonomy) and listed alphabetically. Conceptualization was performed by G.V. Data curation was provided by A.G. Formal analysis was provided by A.G., R.K., and G.V. Funding acquisition was provided by G.V. Investigation was performed by A.G. Methodology was performed by A.G., R.K., and G.V. Project administration was provided by G.V. Resources were provided by G.V. Software was provided by A.G. and G.V. Supervision was done by G.V. Validation was provided by A.G. and G.V. Visualization was done by A.G. Writing – original draft was done by G.V. Writing – review and editing was done by all.

## Supporting information

Supporting Information

Supplemental Movie 1

Supplemental Movie 2

Supplemental Movie 3

## Data Availability

The data that support the findings of this study are available from the corresponding author upon reasonable request.
