# Supplementary material for: Anomalous Dynamics of Superparamagnetic Colloidal Microrobots with Tailored Statistics
Source: Small. 2025 Oct 28;21(47):e06538. doi: 10.1002/smll.202506538 (PMC12658949; doi:10.1002/smll.202506538)
Supplement: Supplementary file 1 — Supporting Information [file SMLL-21-e06538-s001.pdf]

NANO · MICRO
small

Supporting Information

for *Small*, DOI 10.1002/smll.202506538

Anomalous Dynamics of Superparamagnetic Colloidal Microrobots with Tailored Statistics

*Alessia Gentili, Rainer Klages and Giorgio Volpe**

Supporting Information

Anomalous Dynamics of Superparamagnetic Colloidal Microrobots with Tailored Statistics

Alessia Gentili, Rainer Klages, Giorgio Volpe*

**Corresponding author: g.volpe@ucl.ac.uk*

Supporting Text: Stochastic processes in the comoving frame

Here, we provide the theoretical underpinning for our experiments. We first introduce the comoving frame and illustrate its use by a simple example. We then define equations for overdamped stochastic dynamics in it by implementing our experimental constraint of constant speed. Next, we lay out a protocol to define trajectories with constant speed in this reference frame compatible with fractional Brownian motion, which we refer to as *fractional Brownian walks*. Finally, we outline the specific implementation of this protocol for our experiments.

The comoving frame

Stochastic processes are typically defined in a fixed Cartesian frame, as this can simplify their theoretical analysis. In contrast, our experiments are better described by stochastic dynamics formulated in a comoving frame in two dimensions (Figure 1). This is a coordinate frame attached to the center of mass of a moving particle, whose x-axis is aligned with the velocity vector $\mathbf{v}_{n-1}$ at any time step t_{n-1}, and the associated y-axis is perpendicular to it.[72] It is thus corotating with the change of direction of the moving particle, and, hence, includes both translational and rotational movements. In this Cartesian comoving frame the velocity $\mathbf{v}_n$ at the next time step t_n can be expressed in terms of its abscissa $v_{x,n}$ along the x-axis and its ordinate $v_{y,n}$ along the y-axis. However, it is more convenient to formulate the velocity $\mathbf{v}_n$ at the next time step t_n in the comoving frame by its polar coordinates, speed v_n and turning angle φ_n (Figure 1b). The pair (φ_n, v_n) at time steps t_n yields the coordinates of a particle in the comoving frame, and the corresponding time series fully determines its dynamics.

Because of our experimental constant speed constraint, we can directly consider overdamped dynamics in the form of correlated random walks.[42] These are defined by the time-discrete equations of motion in the comoving frame (Equation (1) and (2) in the main text):

$$\varphi_n = \xi_{\varphi,n} \tag{S1}$$

$$v_n = \xi_{v,n} \, . \tag{S2}$$

The terms $\xi_{\varphi,n}$ and $\xi_{v,n}$ represent two noises driving our microrobots' experimental dynamics in this coordinate frame. In principle, these noises can be arbitrarily complex: they can depend on discrete time t_n; they can also be coupled by featuring prefactors that may depend on both state variables; furthermore, they may be correlated in time.

Random walks

To illustrate this general framework, we can consider the two-dimensional time-discrete random walk put forward by Ross and Pearson about a century ago (Figure 1, $\mu = 1$).[73–75] If defined in a Cartesian coordinate frame, we can sample the two velocities, v_x and v_y, along x and y as independently and identically distributed random variables from two corresponding probability distributions, $\xi_{v_x,n} \sim \rho(v_x)$ and $\xi_{v_y,n} \sim \rho(v_y)$, where here both distributions are symmetric Gaussians. Transforming these two velocities into polar coordinates of velocity orientation θ and speed v by using conservation of probability,[76]

$$\rho(v_x)\rho(v_y)dv_x dv_y = \rho(\theta)\rho(v)d\theta dv \, , \tag{S3}$$

yields a uniform distribution on the circle, $\rho(\theta) = (2\pi)^{-1}$, for the corresponding probability density of the orientation and a Rayleigh (two-dimensional Maxwell-Boltzmann) distribution for the corresponding probability density of the speed,

$$\rho(v) = \frac{v}{\sigma_v^2} e^{-\frac{v^2}{2\sigma_v^2}} \ . \tag{S4}$$

where σ_v is the scale parameter of the distribution. After further wrapping on the circle and due to Markovianity, $\rho(\theta)$ delivers an equally uniform turning angle distribution $\rho(\varphi) = (2\pi)^{-1}$ (Figure S5a). Sampling independent and identically distributed random variables from these turning angle and speed distributions according to Equation (S1) and (S2) by converting speeds into step lengths per time interval defines a time-discrete two-dimensional Ross-Pearson random walk in the comoving frame. Alternatively, one could sample the speeds from a (half-)Gaussian, as often done in the literature[44,45] and in our experiments (Figure 2 and S5), which due to the central limit theorem also yields Gaussian position distributions in the long-time limit, like the Ross-Pearson random walk.

Constant speed dynamics

In our experiments, a microrobot moves with a speed that is constant on average, and we can control the flight time τ_n during which it moves ballistically in the same direction (Figure 1). Given some stochastic dynamics with variable speed v_n defined by Equation (S1) and (S2), our goal is to construct a corresponding stochastic process with constant speed that, for clarity of notation, here we denote as $v_\mathrm{c} = \mathrm{const.}$, which exactly preserves the topology of the paths generated by the original variable speed process. That is, a microrobot is supposed to move with constant speed on exactly the same paths generated by exactly the same step-length distribution for both dynamics. This implies that, instead of using constant flight times $\tau_n = \tau_\mathrm{c} = \mathrm{const.}$ for the variable speed dynamics, we use variable flight times τ_n for the constant speed dynamics by preserving the corresponding step lengths ℓ_n for both processes. Using the very same step lengths ℓ_n provides the crucial link between both dynamics. To do so, we need to transform Equation (S2) such that we can sample independently and identically distributed random variables from a distribution of flight times $\xi_{\tau,n} \sim \rho(\tau)$ instead of a speed distribution $\xi_{v,n} \sim \rho(v)$ (Equation (3) in the main text).
We can perform this transformation as follows. Consider a stochastic process for the speed $v_n = \xi_{v,n}$, where the random variable $\xi_{v,n}$ is drawn independently and identically distributed from a given speed distribution $\rho(v)$, $\xi_{v,n} \sim \rho(v)$. Then the distance ℓ_n traveled during a constant flight time τ_c is given by

$$\ell_n = v_n \tau_\mathrm{c} \ . \tag{S5}$$

This defines $\ell_n = \xi_{\ell,n}$ as a new, transformed random variable for which we need to calculate the associated step length distribution $\xi_{\ell,n} \sim \rho(\ell)$. This can be done again by conservation of probability (cf. Equation (S3)), $\rho(v)dv = \rho(\ell)d\ell$, where the Jacobian is calculated from Equation (S5) yielding

$$\rho(\ell) = \frac{1}{\tau_\mathrm{c}} \rho(v) \ . \tag{S6}$$

This completes the transformation from $v_n = \xi_{v,n} \sim \rho(v)$ to the associated stochastic process $\ell_n = \xi_{\ell,n} \sim \rho(\ell)$ at constant flight times τ_c.
Now assume that $v_n = v_\mathrm{c} = \mathrm{const.}$ instead of $\tau_n = \tau_\mathrm{c} = \mathrm{const.}$, as in our experiments (Figure 1 and S2). We wish to preserve the step length ℓ_n at a given discrete time step n, as calculated above, so that the overall topology of a random path is preserved. That is, a microrobot traverses the very same distance ℓ_n for the very same turning angle φ_n at a time step n at now constant speed v_c, paying the price that it will now do so at a typically different (longer or shorter) associated variable flight time τ_n, making up for the corresponding original variable speed v_n. This is exactly the same idea underlying the transformation between Lévy flights and Lévy walks,[3,5] which we implement here within the comoving frame. Accordingly, we now remove the assumption that $\tau_n = \tau_\mathrm{c} = \mathrm{const.}$ in Equation (S5), allowing again for variable flight times τ_n, by implementing instead the constant speed constraint $v_n = v_\mathrm{c} = \mathrm{const.}$ This

leads to the complementary equation

$$\tau_n = \frac{\ell_n}{v_c} , \tag{S7}$$

for the very same ℓ_n as above, where $\tau_n = \xi_{\tau,n}$ is sampled from a flight time distribution $\xi_{\tau,n} \sim \rho(\tau)$. The latter we can obtain from the step length distribution $\rho(\ell)$, Equation (S6), again by conservation of probability. Calculating the involved Jacobian by using Equation (S7) gives

$$\rho(\tau) = v_c \rho(\ell) . \tag{S8}$$

Feeding Equation (S6) into Equation (S8) yields the desired transformation between a given speed distribution at constant flight time τ_c and the corresponding flight time distribution at constant speed v_c,

$$\rho(\tau) = \frac{v_c}{\tau_c} \rho(v) . \tag{S9}$$

The step lengths ℓ_n corresponding to flight times τ_n at constant speed v_c can be obtained from Equation (S7) yielding

$$\ell_n = v_c \xi_{\tau,n} , \tag{S10}$$

which gives Equation (3) in the main text.
As an example of applying this transformation scheme from variable speed at constant flight times to constant speed at variable flight times, consider the Rayleigh distribution for the speed mentioned at the end of the previous section. We wish to transform this distribution into a corresponding flight time distribution by assuming now a constant speed v_c. We can do so by solving Equation (S5) for v_n, solving Equation (S7) for ℓ_n, and replacing ℓ_n in the former equation by the latter yielding

$$v_n = \frac{v_c}{\tau_c} \tau_n . \tag{S11}$$

Applying this change of variables to the stationary Rayleigh speed distribution Equation (S4) yields for the associated stationary flight time distribution at constant speed

$$\rho(\tau) = \frac{\tau}{\sigma_\tau^2} e^{-\frac{\tau^2}{2\sigma_\tau^2}} , \tag{S12}$$

with scale parameter $\sigma_\tau = \tau_c \sigma_v / v_c$, which is also a Rayleigh distribution. An α-stable Lévy speed distribution can be transformed into a corresponding α-stable Lévy flight time distribution exactly along the same lines. Since an exact definition for all parameter values $\alpha = 3 - \mu$ can only be given through its characteristic function in Fourier space, here we focus on the asymptotic representation in the form of power-law tails,[3,5]

$$\rho(v) \approx C_1(\alpha)\, v^{-(\alpha+1)}\, (v \to \infty) \tag{S13}$$

with $C_1(\alpha) = (1/\pi) \sin(\pi\alpha/2)\Gamma(1+\alpha)$. Applying Equation (S11) then gives

$$\rho(\tau) \approx C_1(\alpha) \frac{v_c}{\tau_c}\, \tau^{-(\alpha+1)}\, (\tau \to \infty) , \tag{S14}$$

yielding the functional form of Equation (9), which was used for the experiments.
We emphasize that, while this general transformation between variable and constant speed is designed to preserve the topology of a given random path by preserving the step length generated at any discrete time step, it strictly yields a pair of variable- and constant-speed stochastic processes that are *not equivalent* to each other, see Equation (1) and (2) and Equation (1) and (3) in the main text. Clearly, for the former the speed distribution is not a δ-function while for the latter, by definition, it is, and vice versa for the corresponding flight time distributions. This non-trivial connection and its consequences for the corresponding stochastic properties have been amply explored in the literature, e.g., for Lévy flights and walks.[3,5]
Finally, choosing for $\xi_{\varphi,n} \sim \rho(\varphi)$ in Equation (1) a uniform distribution and for $\xi_{\tau,n} \sim \rho(\tau)$ in Equation (3) a half-Gaussian distribution yields normal diffusion, while choosing for $\xi_{\varphi,n} \sim \rho(\varphi)$ in Equation 1 a uniform distribution and for $\xi_{\tau,n} \sim \rho(\tau)$ in Equation (3) an α-stable Lévy distribution defines a uniform Lévy walk in the comoving frame (Figure 1, 2 and 3).[43]

Fractional Brownian motion and walks

The previous transformation between variable-speed and constant-speed dynamics applies to Markovian stochastic processes, i.e. without memory between different steps ℓ_n. In our experiments, we also want to generate spatiotemporally correlated dynamics. A paradigmatic example of such a process is fractional Brownian motion (FBM). Therefore, in this section, we first discuss how to generate an analogue of two-dimensional FBM in a comoving frame under the constraint of constant speed to then explain our specific experimental implementation (Figure 4) in the following section (*Experimental implementation of fractional Brownian walks*). FBM is a Gaussian power-law correlated stochastic process that generates the whole spectrum of anomalous diffusion under parameter variation, from subdiffusion to superdiffusion through normal diffusion.[7,8] While Lévy walks yield superdiffusion due to sampling the step lengths ℓ_n from power-law distributions instead of Gaussians (Figure 1 and 2), FBM defines a fundamentally different class of anomalous stochastic process, where anomalous diffusion is due to a non-Markovian, power-law correlation decay of the velocity autocorrelation function (VACF) while sampling the velocities from Gaussian distributions.[77,78]
FBM is a continuous process in space and time, which can be defined in terms of overdamped Langevin dynamics driven by fractional Gaussian noise (FGN).[8,49] In one dimension, the corresponding equation reads

$$\frac{dx}{dt} = \xi_{\mathrm{FGN}}(t)\,, \tag{S15}$$

where $\xi_{\mathrm{FGN}}(t)$ holds for fractional, i.e. power-law correlated Gaussian noise[8,49]

$$\langle \xi_{\mathrm{FGN}}(t_1)\xi_{\mathrm{FGN}}(t_2)\rangle = 2K_H H\Big[(2H-1)|t_1-t_2|^{2H-2} + 2|t_1-t_2|^{2H-1}\delta(t_1-t_2)\Big] \tag{S16}$$

with Hurst exponent $0 < H < 1$ and generalized diffusion coefficient K_H. The correlation function of the noise is thus identical with the VACF of the FBM process, which decays as a power law in time. From this equation, the mean squared displacement of FBM can be calculated to[8,49]

$$\langle x^2(t)\rangle = 2K_H t^{2H}\,. \tag{S17}$$

Under variation of the power-law exponent $\mu = 2H$, FBM displays the whole spectrum of anomalous diffusion.
For our experiments, we need to define a process at constant speed in the comoving frame that preserves the topology of the paths generated by FBM. Since, to our knowledge, no definition of such a stochastic process exists in this frame, we design a protocol to perform the required transformation. In line with our experimental set-up, we define this protocol in terms of time- and space-discrete FBM.[8],[79] The crucial aspect for this transformation is to capture the non-Markovian correlations in Equation (S16) inherent to FBM. To do so, we consider a whole time series of a time-discrete FBM path (e.g. as generated in simulations) by recording the exact sequence of speeds and associated turning angles (v_n, φ_n). We then use our change of variables in Equation (S11) to generate a Cartesian constant-speed version of FBM, defining the corresponding sequence of flight times and associated turning angles (τ_n, φ_n). In addition to preserving the step lengths at any time step n, reproducing the velocity correlations requires us to also preserve the turning angles between pairs of velocities at subsequent n. Using the resulting full sequence of flight times and turning angles (τ_n, φ_n) at constant speed indeed enables us both to maintain the initial correlation (as we show below) and to reproduce the topology of the original variable speed FBM in the comoving frame. We can consider this resulting constant-speed analogue of FBM as a *fractional Brownian walk*, in analogy to transforming Lévy flights into Lévy walks.[5] The detailed protocol achieving this transformation from variable-speed to constant-speed FBM is defined as follows:

1. Generate two-dimensional time-discrete Cartesian FBM by a standard numerical algorithm.

2. Extract the sequence of speeds v_n and turning angles φ_n from a path of this numerically generated FBM at any time step $t_n = n\tau_{\mathrm{c}}$ for given $\tau_{\mathrm{c}} = \mathrm{const.}$

3. For a given v_n in the sequence, calculate a corresponding flight time τ_n according to Equation (S11) by setting the desired speed $v_\mathrm{c} = \mathrm{const.}$

4. Reproduce the sequence of (τ_n, φ_n) pairs in the comoving frame with constant speed v_c, thus obtaining a constant-speed analogue of FBM in this frame that can be implemented with Equation (1) and (3).

The velocities of this process are no longer Gaussian-distributed nor are the speeds Rayleigh-distributed. However, according to Equation (S11), we can expect the flight-time distribution of this process at constant speed (corresponding to a variable-speed Gaussian stochastic process like FBM at constant flight times) to be Rayleigh-distributed (Equation (S12)), as confirmed experimentally (Figure S12). Most importantly, we expect this FBM-like process to still preserve the non-Markovian VACF decay (Equation (S16)) as a defining feature of the original Gaussian FBM process. The latter property can be verified by starting from the definition of the VACF for an arbitrary non-constant speed, two-dimensional time-discrete process, given by

$$\langle \mathbf{v}_0 \mathbf{v}_n \rangle = \langle v_0 v_n \cos\varphi_n \rangle \, , \tag{S18}$$

where, for $\mathbf{v}_0 \parallel x$, the angle φ_n is the polar angle between the velocity vector $\mathbf{v}_0$ at time step $n = 0$ and the velocity vector $\mathbf{v}_n$ at time step n, with the angular brackets defining a suitable (time or ensemble) average. Assuming that the initial speed $v_0 = \mathrm{const.}$, we can rewrite

$$\langle \mathbf{v}_0 \mathbf{v}_n \rangle = v_0 \langle v_n \cos\varphi_n \rangle \, . \tag{S19}$$

As the polar coordinates, speed v_n and orientation φ_n, for the velocity vector $\mathbf{v}_n$ are decoupled,

$$\langle \mathbf{v}_0 \mathbf{v}_n \rangle = v_0 \langle v_n \rangle \langle \cos\varphi_n \rangle \, . \tag{S20}$$

Because, by definition, our protocol preserves all angles φ_n of the original time-discrete FBM process, we conclude that the VACF of the underlying original FBM process we started from is preserved if the constant speed $v_\mathrm{c} = v_0$ fulfills the equation

$$v_\mathrm{c} = \langle v_n \rangle \, . \tag{S21}$$

For the speed-constant process, by averaging over Equation (S11) and using Equation (S21) we furthermore obtain

$$\tau_\mathrm{c} = \langle \tau_n \rangle \, . \tag{S22}$$

This relationship provides an important matching of timescales, indicating that the decay of the original FBM VACF can only be observed at times longer than the cutoff time defined by τ_c.

Experimental implementation of fractional Brownian walks

In our protocol above, we first need to generate FBM via a standard numerical algorithm, e.g. via the Davies-Harte method as done in our case.[67] However, the associated discretization of FBM[79] generates spurious dependencies on the Hurst exponent H, which propagate into this method.[67] To avoid introducing such dependencies, we initially simulated space-time-discrete FBM via the Davies-Harte algorithm setting the constant flight time ($\tilde{\tau}_\mathrm{c}$) and the scale parameter ($\sigma_{\tilde{v}}$) to unitary values instead of directly setting the values needed for our experiments. Note that we now denote all variables generated in simulations with a tilde while all experimentally reproduced ones are still written without it. To match our experimental length scales, we then rescaled the distance $\tilde{\ell}_n$ traveled in simulations during the constant flight time $\tilde{\tau}_\mathrm{c}$ by a factor κ, thus giving the actual experimental step length ℓ_n for our microrobots as

$$\ell_n = \kappa \tilde{\ell}_n \, . \tag{S23}$$

We can therefore redefine all the relevant quantities discussed in the previous sections by taking this scaling into account. For brevity, in the following we just give the main results. After rescaling, Equation (S5) yields

$$v_n = \frac{\kappa \tilde{\ell}_n}{\tilde{\tau}_\mathrm{c}} \tag{S24}$$

with scaled speed

$$v_n = \kappa \tilde{v}_n \ . \tag{S25}$$

Applied to Equation (S4), we obtain the scaled stationary Rayleigh speed distribution

$$\rho(v) = \frac{v}{\sigma_v^2} e^{-\frac{v^2}{2\sigma_v^2}} \tag{S26}$$

with scale parameter $\sigma_v = \kappa \sigma_{\tilde{v}}$. Next, the scaled Equation (S7) reads

$$\tau_n = \frac{\kappa \tilde{\ell}_n}{\tilde{v}_\mathrm{c}} \tag{S27}$$

with scaled flight time

$$\tau_n = \kappa \tilde{\tau}_n \ . \tag{S28}$$

Combining Equation (S24) and (S27) leads to the scaled change of variables

$$v_n = \frac{\tilde{v}_\mathrm{c}}{\tilde{\tau}_\mathrm{c}} \tau_n \ . \tag{S29}$$

With this, we obtain the scaled Rayleigh flight time distribution

$$\rho(\tau) = \frac{\tau}{\sigma_\tau^2} e^{-\frac{\tau^2}{2\sigma_\tau^2}} \ , \tag{S30}$$

with scale parameter $\sigma_\tau = \tilde{\tau}_\mathrm{c} \sigma_v / \tilde{v}_\mathrm{c} = \kappa \sigma_{\tilde{\tau}}$. Note that this distribution is defined with respect to the original parameters $\tilde{\tau}_\mathrm{c}$ and $\tilde{v}_\mathrm{c}$ used in the simulations before scaling. Along the same lines, the scaled VACF is now given by

$$\langle \mathbf{v}_0 \mathbf{v}_n \rangle = \kappa^2 \langle \tilde{\mathbf{v}}_0 \tilde{\mathbf{v}}_n \rangle \ . \tag{S31}$$

Following the same reasoning as before, we choose

$$v_\mathrm{c} = \kappa \tilde{v}_\mathrm{c} = \kappa \tilde{v}_0 \tag{S32}$$

yielding, as a condition for the scaled constant-speed FBM dynamics to preserve the topology of the original FBM process,

$$v_\mathrm{c} = \kappa \langle \tilde{v}_n \rangle = \langle v_n \rangle \tag{S33}$$

as the scaled analogue to Equation (S21). Likewise, for the relevant timescale of the scaled constant-speed process, we obtain

$$\tau_\mathrm{c} = \kappa \tilde{\tau}_\mathrm{c} = \kappa \langle \tilde{\tau}_n \rangle = \langle \tau_n \rangle \tag{S34}$$

as the scaled analogue to Equation (S22). As before, Equation (S34) provides an important matching of timescales, indicating that the decay of the original FBM VACF can only be observed at times longer than the scaled cutoff time defined by $\kappa \tilde{\tau}_\mathrm{c}$. In our experiments, for sampling times δt below this cut-off time, one can indeed observe the typical exponentially decaying short-time correlation due to the microrobots' short-term drift in the magnetic driving field.[23]

Supporting Figures

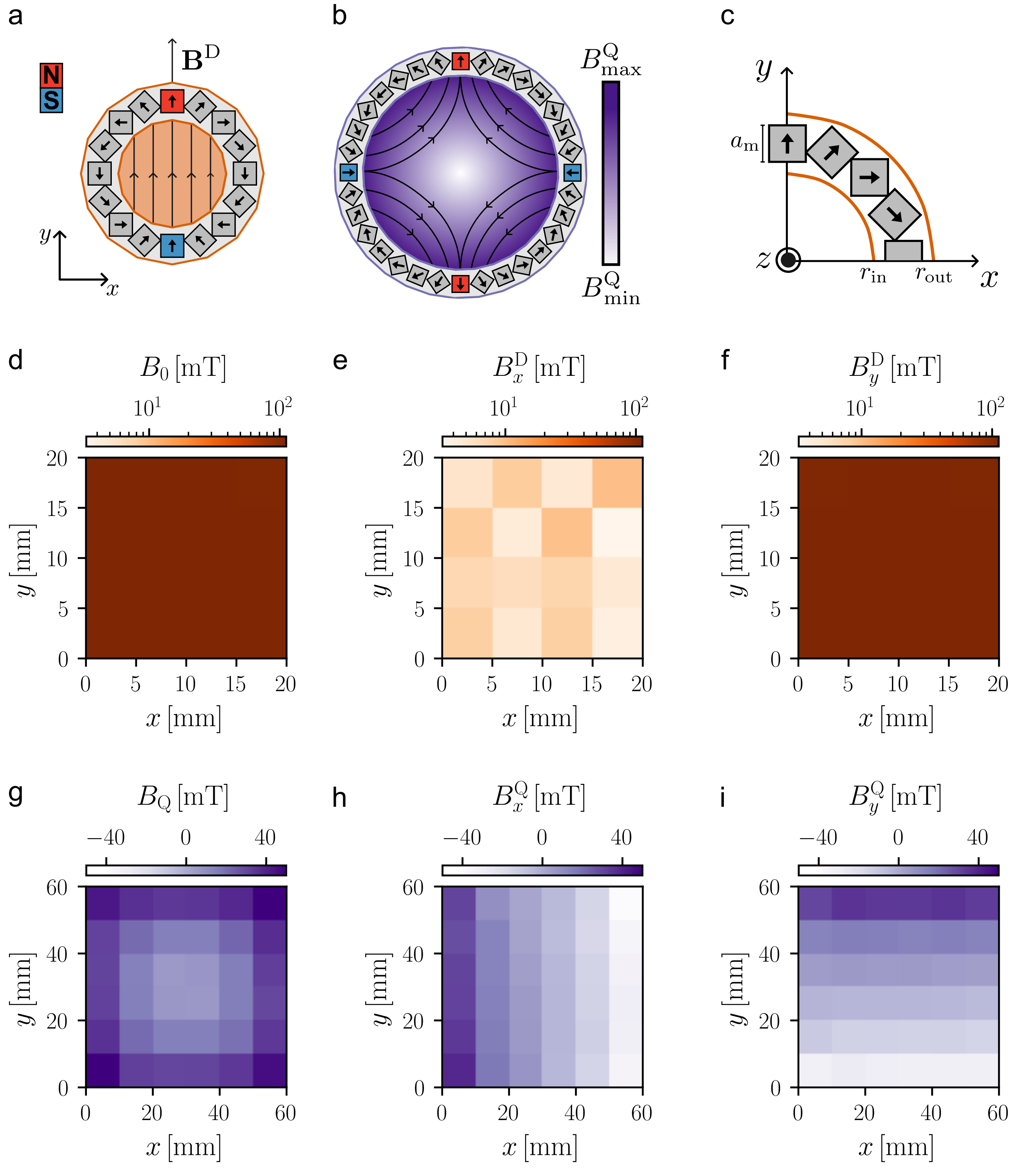

Figure S1: Magnetic fields of the Halbach cylinders. (a-b) In-scale schematics of a cylindrical Halbach (a) dipole and (b) quadrupole, built with cubic permanent magnets (gray squares with black arrows pointing to their north poles). The cylinders' magnetic north (N, red) and south (S, blue) poles as well as flux lines are shown. The strong dipolar field $\mathbf{B}^{\mathrm{D}} = B_0\mathbf{e}_y$ is homogeneous and directed along the y-axis (unit vector, $\mathbf{e}_y$). The weaker quadrupolar field $\mathbf{B}^{\mathrm{Q}}$ consist of two orthogonal linear components. (c) The cubic magnets (side length, a_{m}) are arranged in a circular pattern in the xy plane, with the cylinders' axis along z (Experimental Section, Table S1). The inner radius r_{in} (from the center to the magnets' inner edge) defines the experimental area. The outer radius r_{out} (from center to the magnets' outer edge) delimits the size of the cylinder. (d) Measured dipolar magnetic field intensity B_0 with (e) x- and (f) y-components, B^{D}_x and B^{D}_y. B^{D}_x is one order of magnitude smaller than B^{D}_y. (g) Measured quadrupolar magnetic field intensity B_{Q} with (h) x- and (i) y-components, B^{Q}_x and B^{Q}_y, which are orthogonal and linear in space.

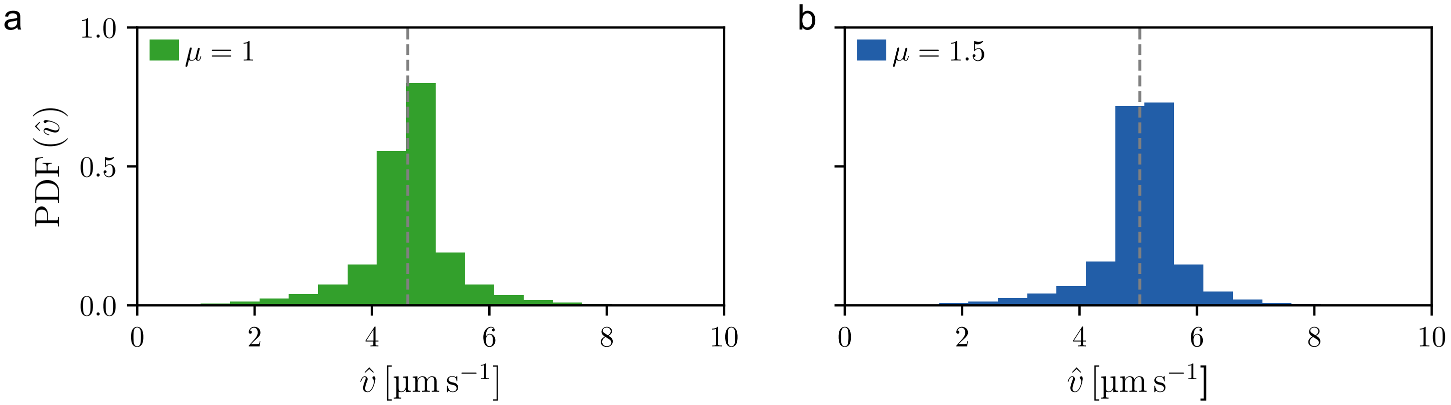

Figure S2: Instantaneous microrobots' speed. (a-b) Probability density functions (PDF) of the instantaneous microrobots' speed $\hat{v}$ extracted from the two trajectories in Figure 1e for the cases of (a) normal diffusion ($\mu = 1$) and (b) superdiffusion ($\mu = 1.5$). The speed was calculated using a time window of 0.42 s (corresponding to five video frames) moving along the trajectory to minimize the impact of the tracking localization noise. The average speed (dashed vertical lines) is (a) $\langle \hat{v} \rangle = 4.61 \pm 0.82$ µm s^{-1} and (b) $\langle \hat{v} \rangle = 5.03 \pm 0.74$ µm s^{-1}. Due to the narrow distributions, we consider the microrobots to move approximately at constant speed in our experiments.

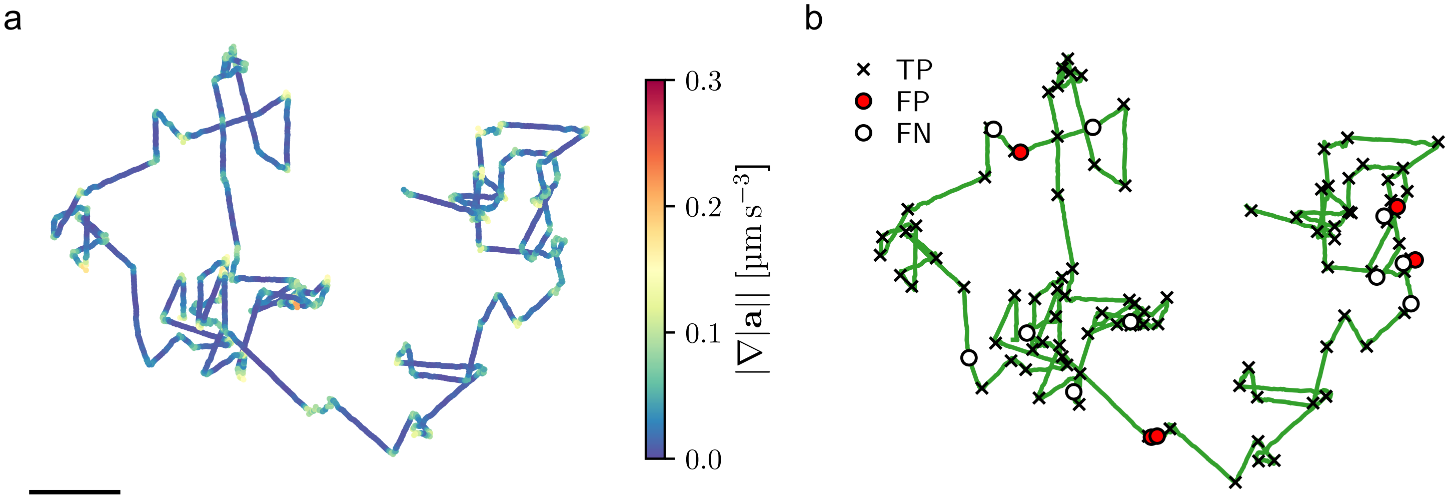

Figure S3: Detection of turning points on microrobots' trajectories. (a) Portion of the normal diffusion trajectory in Figure 1e with the absolute values of the acceleration magnitude gradient ($|\nabla|\mathbf{a}||$) superimposed. Peaks in this quantity identify the turning points (Experimental Section). (b) The detected turning points are validated against the quadrupole rotations by calculating the F1 score from true positives (crosses, TP), false positives (filled circles, FP), and false negatives (empty circles, FN) (Experimental Section). Scale bar: 25 µm.

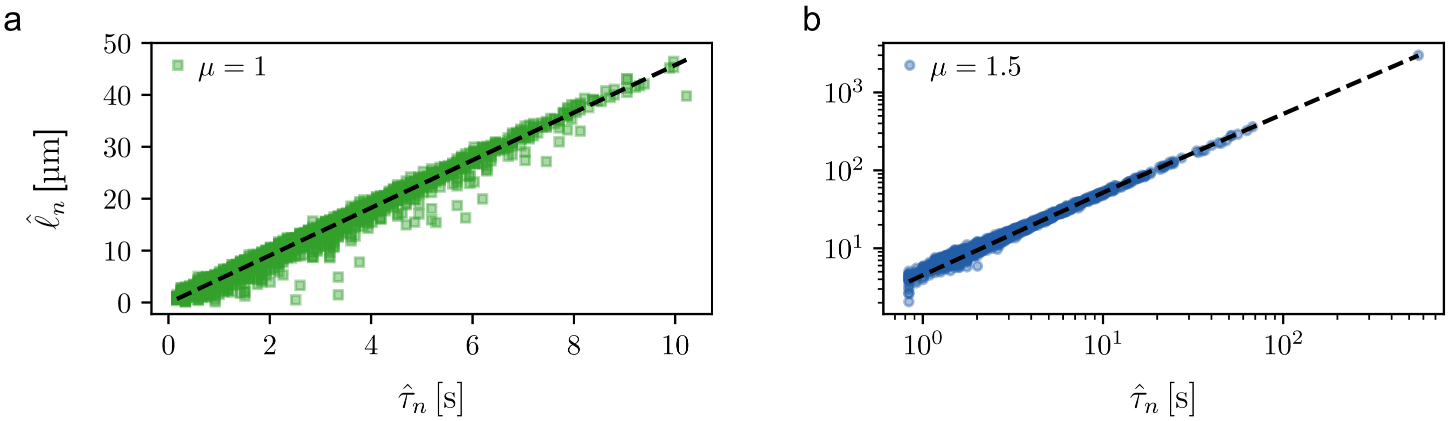

Figure S4: Linearity between step lengths and flight times. Microrobots' step lengths $\hat{\ell}_n$ as a function of flight times $\hat{\tau}_n$ between turns extracted from the two trajectories in Figure 1e for the cases of (a) normal diffusion ($\mu = 1$) and (b) superdiffusion ($\mu = 1.5$). The linear fits to the data (black dashed lines) confirms that the microrobots move at an approximately constant speed for all step lengths. The slope of the fit lines provides an estimate of the average speed of the microrobots alternative to the instantaneous velocity (Figure S2): (a) $\langle \hat{v} \rangle = 4.585 \pm 0.009$ μm s^{-1} and (b) $\langle \hat{v} \rangle = 5.298 \pm 0.002$ μm s^{-1}. These values are in good agreement with those in Figure S2.

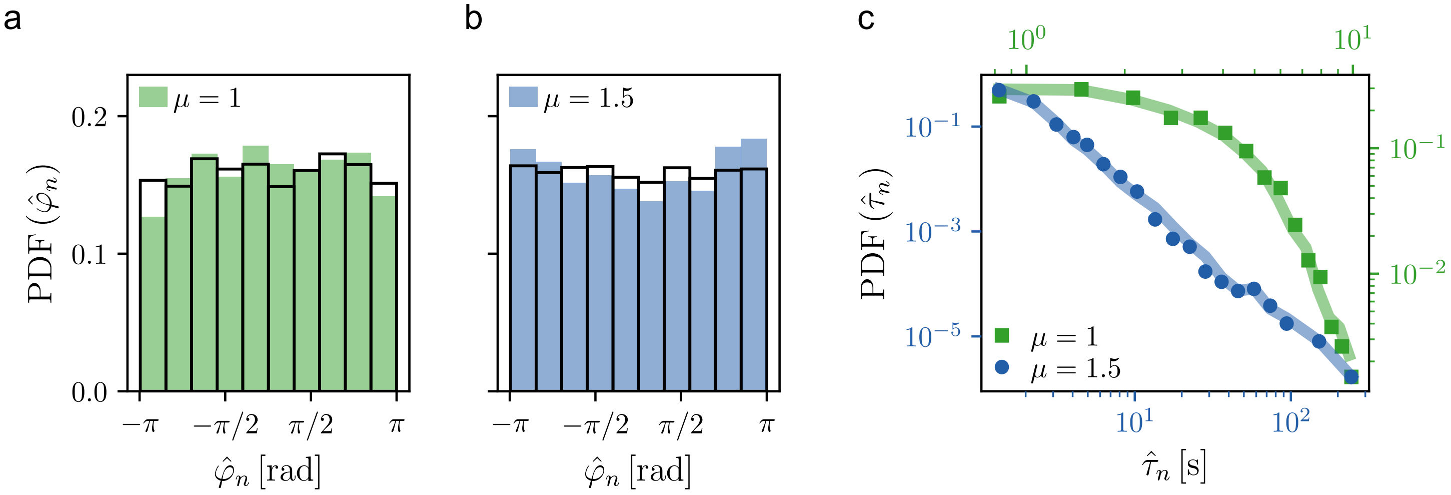

Figure S5: Distributions of microrobots' turning angles and flight times. (a-b) Probability density functions (PDF) of the turning angles $\hat{\varphi}_n$ extracted from the two individual trajectories in Figure 1e for (a) normal diffusion ($\mu = 1$) and (b) superdiffusion ($\mu = 1.5$). The PDFs of twice the quadrupole rotation angles ($\varphi_n = 2\Delta\beta$) are represented with black histograms in (a-b), indicating uniform distributions on the circle and that PDF($\hat{\varphi}_n$) $\sim$ PDF(φ_n) = PDF($2\Delta\beta$) (Experimental Section). (c) PDFs of microrobot's flight times $\hat{\tau}_n$ (as in Figure 2a) for the trajectories in Figure 1e corresponding to normal diffusion ($\mu = 1$, green squares) and superdiffusion ($\mu = 1.5$, blue circles). The thick background lines are the PDFs of the quadrupole rotation times τ_n, showing that PDF($\hat{\tau}_n$) $\sim$ PDF(τ_n). The PDFs of the superdiffusive case are calculated from three individual trajectories for an increased statistical content of their tails. The axis colors reflect those of the respective distributions.

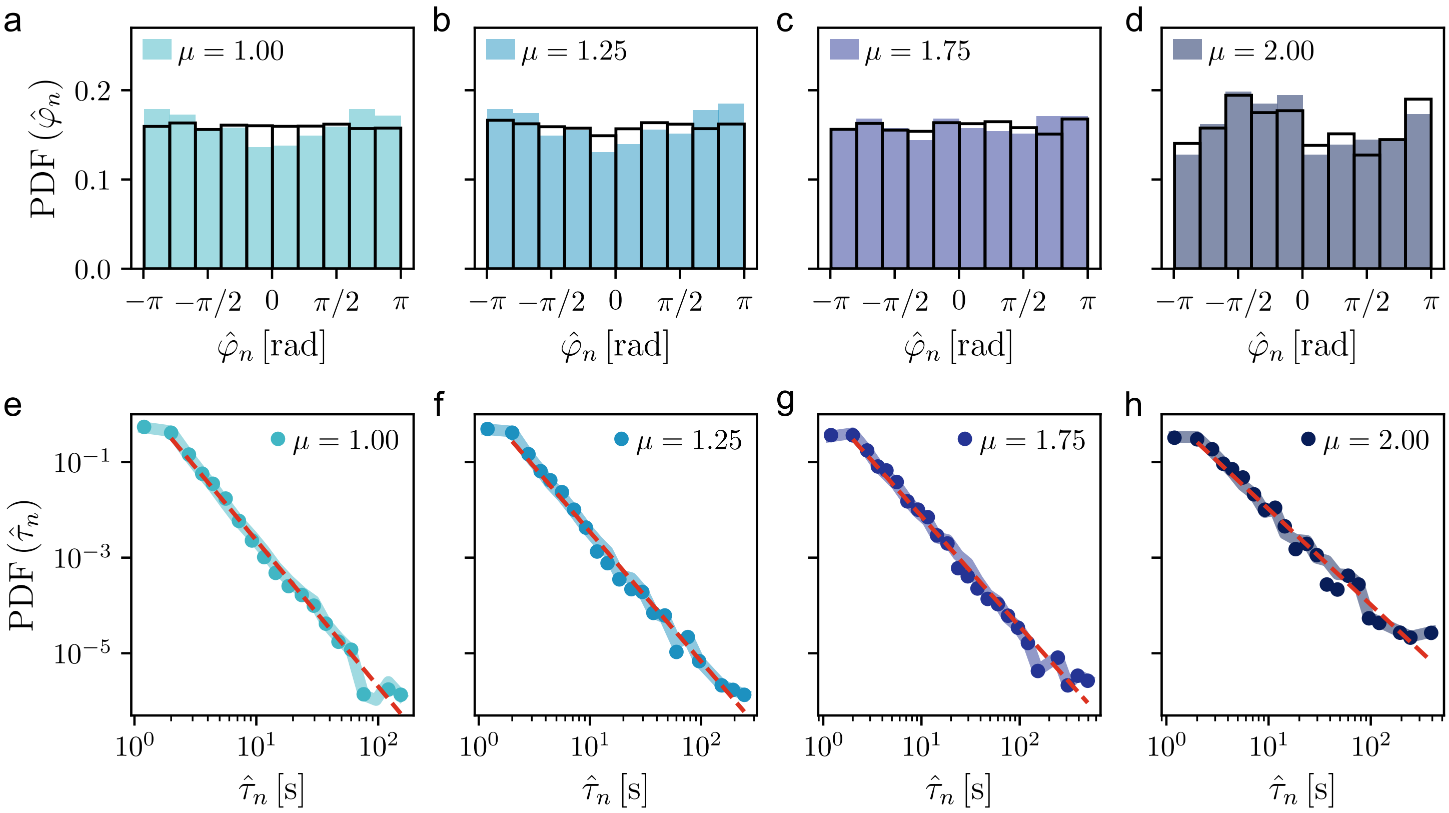

Figure S6: Distributions of microrobots' turning angles and flight times for Lévy walks. Probability density functions (PDF) of (a-d) microrobots' turning angles $\hat{\varphi}_n$ and (e-h) flight times $\hat{\tau}_n$ corresponding to the trajectories in Figure 3a yielding Lévy walks with different anomalous diffusion exponents μ. In (a-d), the PDFs of twice the quadrupole rotation angles ($\varphi_n = 2\Delta\beta$) are represented with black histograms, indicating uniform distributions on the circle and that PDF$(\hat{\varphi}_n) \sim$ PDF$(\varphi_n) =$ PDF$(2\Delta\beta)$ (Experimental Section). In (e-h), the PDFs are calculated from three individual trajectories for an increased statistical content of their tails and the data are fitted to power laws (red dashed lines) showing consistent scaling ($\sim \hat{\tau}_n^{\hat{\mu}-4}$) with Lévy walk models (Table S2). The thick background lines are the PDFs of the quadrupole rotation time τ_n, showing that PDF$(\hat{\tau}_n) \sim$ PDF(τ_n).

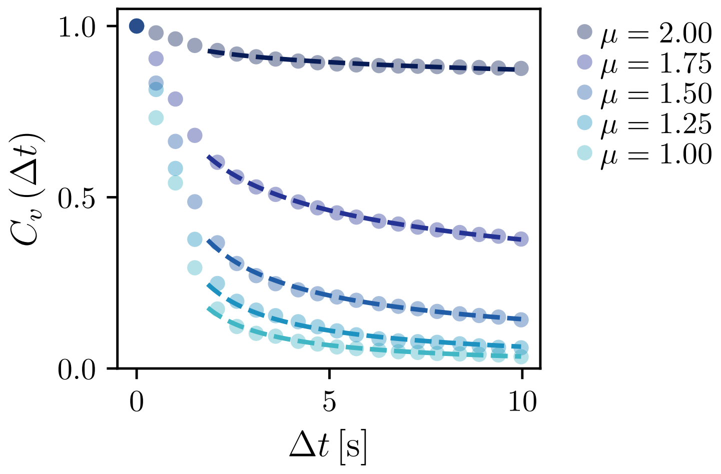

Figure S7: Velocity autocorrelation function for Lévy walks. Normalized time-averaged velocity autocorrelation C_v as a function of lag time Δt for the individual trajectories in Figure 3a, yielding Lévy walks with different anomalous diffusion exponents μ. Fitting the tail of the data with a power law (dashed lines) confirms the scaling characteristic of Lévy walks ($\sim \Delta t^{\hat{\mu}-2}$, Table S2).

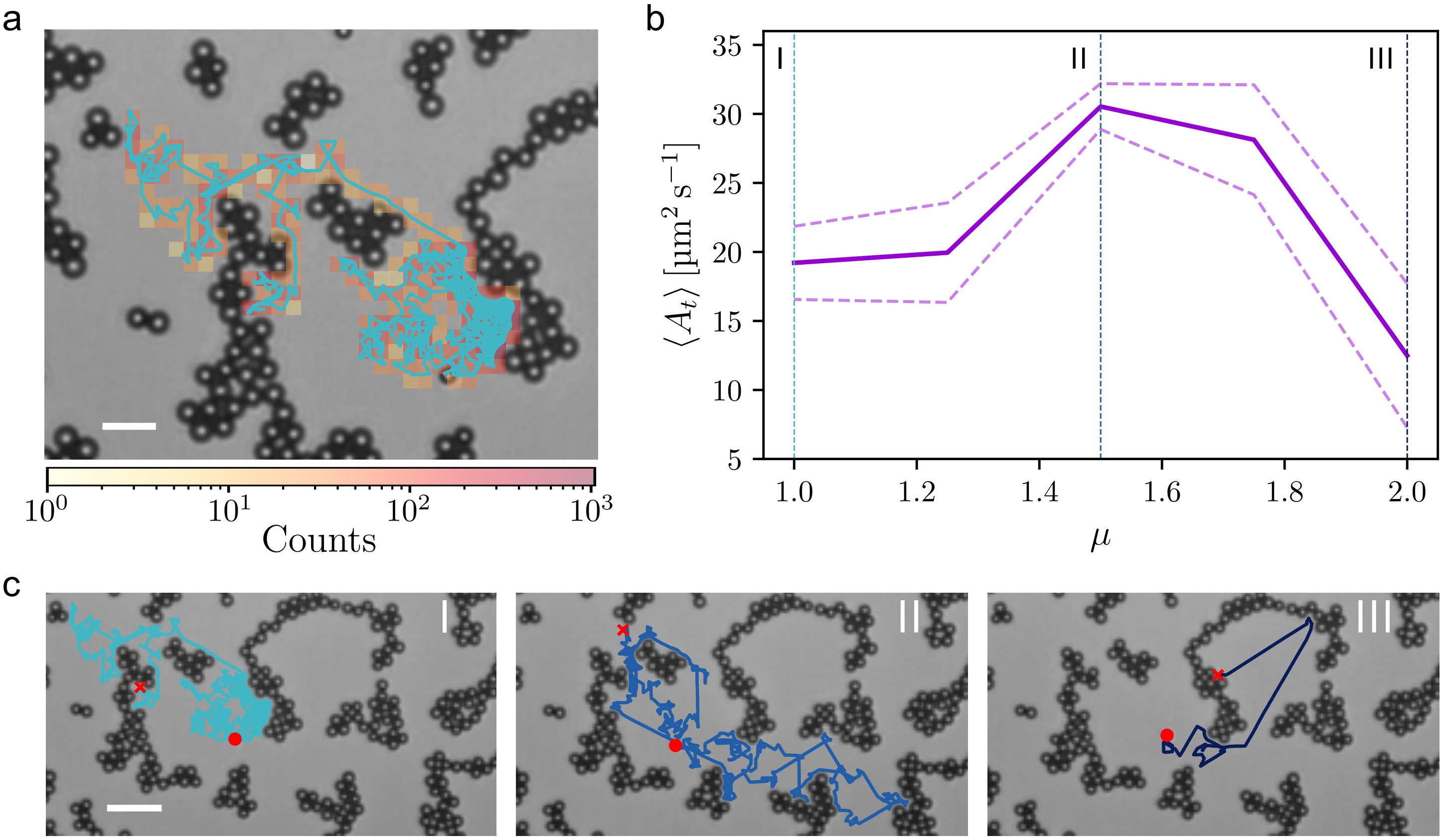

Figure S8: Lévy walks in a complex environment. (a) Exemplary trajectory of a microrobot performing a Lévy walk with $\mu = 1$ on a surface with fixed micro-obstacles (SiO_2 particles of diameter 20.00 ± 0.64 µm). The histogram (with a bin equal to the microrobot's diameter, see Experimental Section) shows the times (counts, colorbar) that the microrobot was found at a given site in the field of view, highlighting an increased permanence in the proximity of the obstacles. Scale bar: 50 µm. (b) Average area exploration rate $\langle A_t \rangle$ as a function of the anomalous diffusion exponent μ. A_t is defined as the area of the unique visited sites (the sum of the areas of the histogram bins in (a) that are occupied at least once) normalized by the trajectory duration. The dashed lines represent the standard error from four independent trajectories per value of μ. (c) Representative trajectories with start (red dot) and end (red cross) points for the three values of μ highlighted by vertical dashed lines in (b): (I) $\mu = 1$, (II) $\mu = 1.5$ where $\langle A_t \rangle$ peaks, and (III) $\mu = 2$. Scale bar: 100 µm.

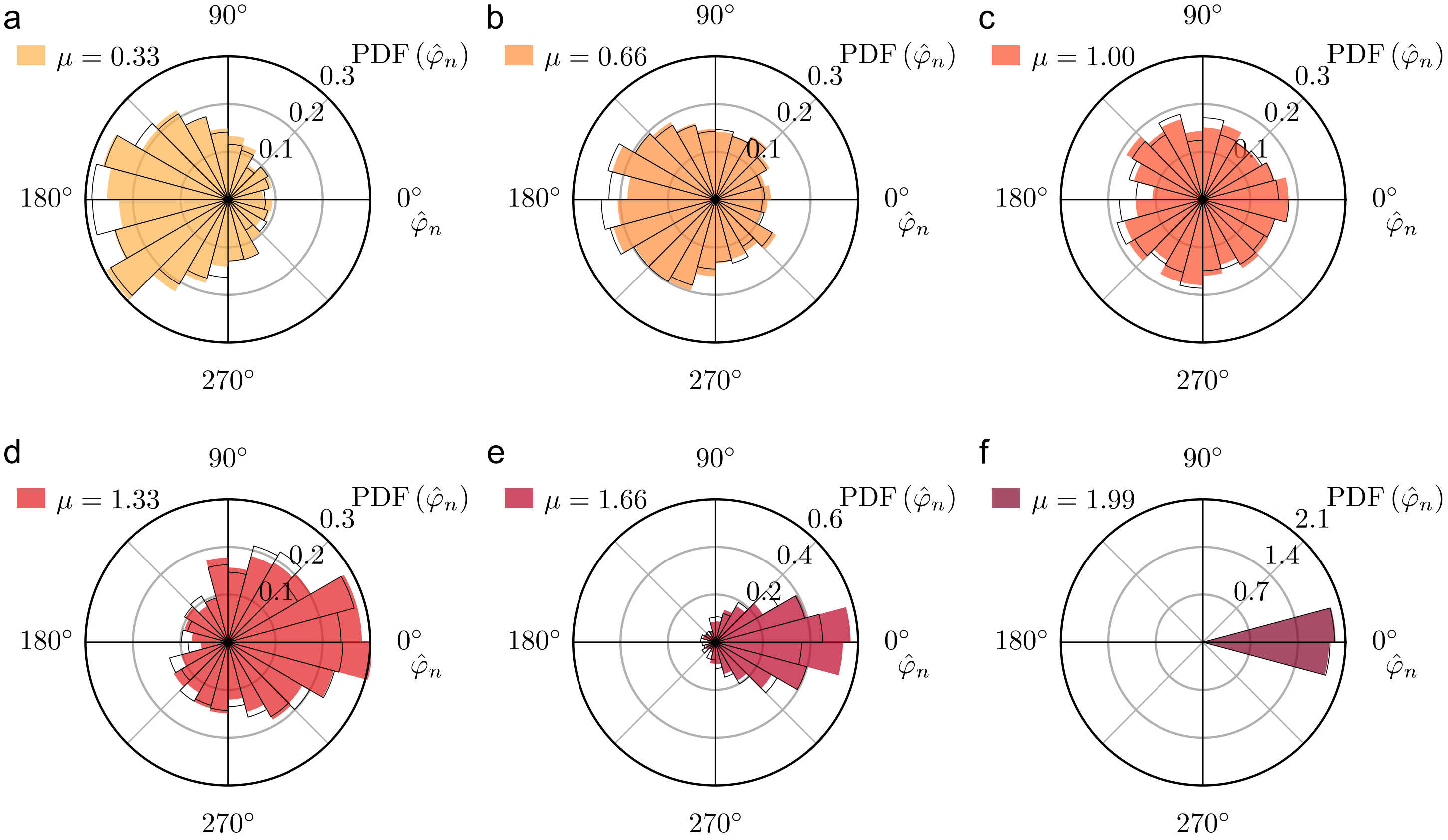

Figure S9: Distributions of turning angles for fractional Brownian walks. Polar plots showing the probability density function (PDF, radial values) of the microrobots' turning angles $\hat{\varphi}_n$ (angular values) from the individual trajectories in Figure 4a showing the shift from backward (negative persistence) to forward propagation (positive persistence) as the anomalous diffusion exponent μ increases from subdiffusion (a: $\mu = 0.33$; b: $\mu = 0.66$) to diffusion (c: $\mu = 1$) to superdiffusion (d: $\mu = 1.33$; e: $\mu = 1.66$; f: $\mu = 1.99$). (a-b) In subdiffusive motion ($\mu < 1$), microrobots tend to move in a direction opposite to their previous one (towards 180°). This tendency is stronger the lower the values of μ. (c) For normal diffusion ($\mu = 1$), the uniform distribution of turning angles highlights the absence of directional correlations. (d-f) In superdiffusive regimes ($\mu > 1$), the microrobots are likely to maintain their current direction (towards 0°), as in directed motion. This tendency is stronger the higher the values of μ. The black solid lines represent the distributions of twice the quadrupole rotation angle for reference.

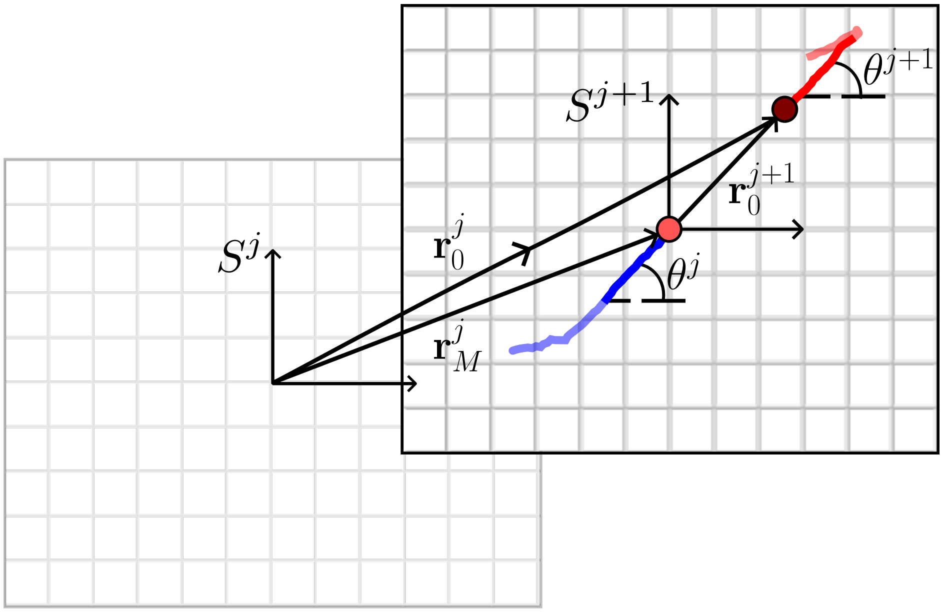

Figure S10: Trajectory stitching. Vectorial relationship (Equation (7)) for the translation of the trajectory points $\mathbf{r}_i^{j+1}$ (red) acquired in the coordinate systems S^{j+1} of the $(j+1)^{\text{th}}$ video back to points $\mathbf{r}_i^j$ (blue) in the coordinate system S^j of the jth video to reconnect them to the previous part of the full microrobot's trajectory. $\mathbf{r}_M^j$ is the position of the last recorded point in the jth video in its reference system, and $\mathbf{r}_0^{j+1}$ and $\mathbf{r}_0^j$ respectively identify the first microrobot's position in the $(j+1)^{\text{th}}$ video in its reference system and in the reference system of the jth video. To facilitate interpolation of the stitched trajectory, any two consecutive videos respectively finished (dark blue line) and started (dark red line) with an at least 1-s long portion of the same step length in the full trajectory, with polar angles θ^j and θ^{j+1} in each respective reference system. The side length of each grid square corresponds to 5 µm.

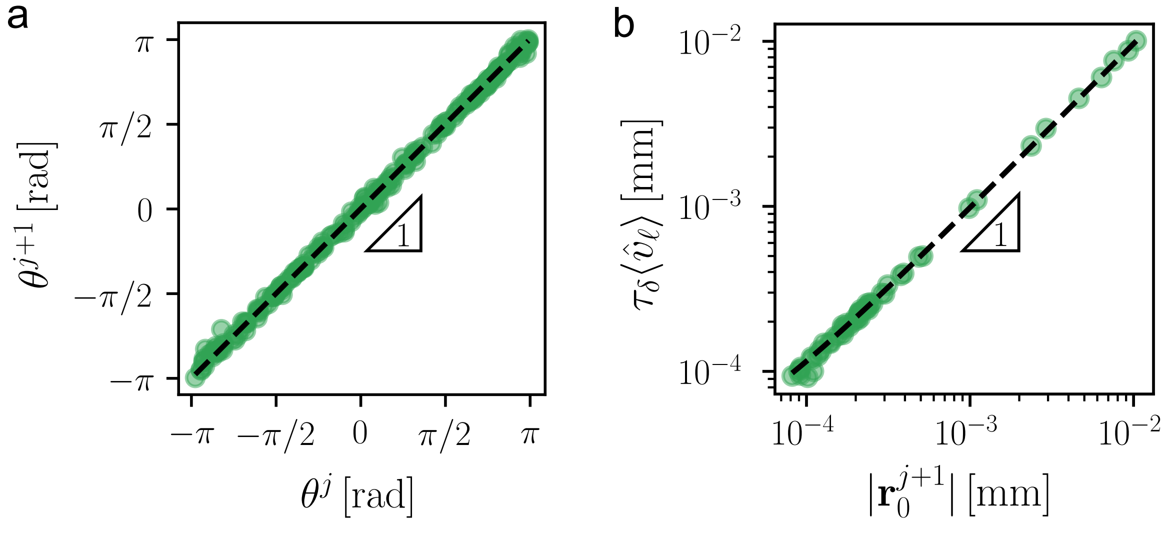

Figure S11: Verification of trajectory stitching. (a) Linear relationship between θ^j and θ^{j+1} (as defined in Figure S10) showing that $\theta^{j+1} \approx \theta^j$. A linear fit to $f(\theta^j) = k'\theta^j$ (dashed line) with slope $k' = 1.002 \pm 0.002$ confirms that the microrobot's direction of motion is preserved during the reconstruction of the trajectory. (b) Linear relationship between direct measurements of the distance $|\mathbf{r}_0^{j+1}|$ (as defined in Figure S10) from image analysis and measurements of stage displacement, and its estimate as $\tau_\delta\langle\hat{v}_\ell\rangle$, where $\langle\hat{v}_\ell\rangle$ is the mean microrobot's speed in the two reconnected portions from the same step length and τ_δ is the time elapsed between recordings of two consecutive videos. A linear fit to $f(|\mathbf{r}_0^{j+1}|) = k'|\mathbf{r}_0^{j+1}|$ with slope $k' = 0.965 \pm 0.003$ confirms the good match between these two values. Linear trend with slope 1 shown for reference.

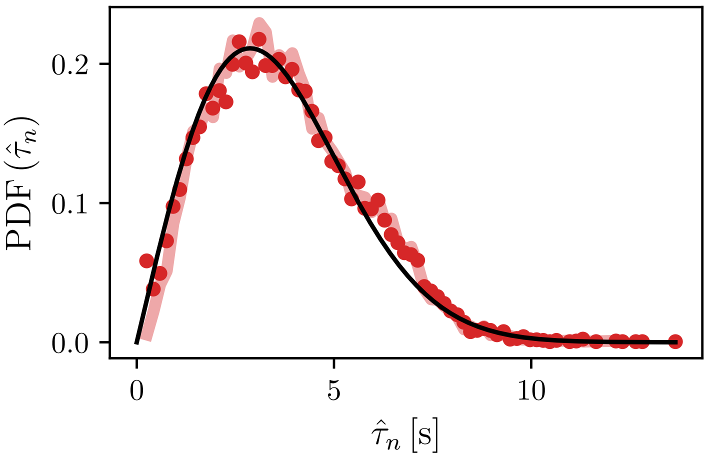

Figure S12: Flight time distribution for fractional Brownian walks. Probability density function (PDF) of microrobots' flight times ($\hat{\tau}_n$) for all fractional Brownian walks (Figure 4a). Data collapse on a Rayleigh distribution given by Equation (S30) (black solid line), independent of the value of the anomalous diffusion exponent μ. The thick background line is the PDF of the quadrupole rotation time τ_n, showing that PDF($\hat{\tau}_n$) $\sim$ PDF(τ_n).

Supporting Tables

Table S1: Parameters of the Halbach cylinders. Remanence B_R and relative permeability μ_R of k cubic neodymium magnets with side length a_m used to build two identical circular arrays for the Halbach dipole and one for the Halbach quadrupole for the experiments. The Halbach cylinders are characterized by the following parameters: inner radius r_{in}, outer radius r_{out}, average radius r_c and height $h = a_m$.

Halbach cylinder	k	B_R [T]	μ_R	a_m [mm]	r_{in} [mm]	r_{out} [mm]	r_c [mm]	h [mm]
Dipole	16	1.48	1.05	9.5	23.3	36.8	30.05	9.5
Quadrupole	32	1.32	1.05	7.0	41.8	51.6	46.7	7.0

Table S2: Anomalous diffusion exponent of Lévy walks. Estimated anomalous diffusion exponent $\hat{\mu}$ for Lévy walks (Figure 2 and 3) based on fitting the mean squared displacement ($\hat{\mu}_{MSD}$, Figure 1 and 3), the quadrupole rotation time probability distribution function ($\hat{\mu}_{\tau_n}$, Figure 2, Figure S5 and S6), the flight time probability distribution function ($\hat{\mu}_{\hat{\tau}_n}$, Figure 2, Figure S5 and S6), the step length probability distribution function ($\hat{\mu}_{\hat{\ell}_n}$, Figure 2 and 3), and the velocity autocorrelation function ($\hat{\mu}_{C_v}$, Figure S7). The first column shows the corresponding ground truth value of μ. Corresponding instantaneous microrobot's speed $\langle \hat{v} \rangle$ are also shown. Errors represent one standard deviation of the respective fit parameters.

μ	$\langle \hat{v} \rangle$ [µm s^{-1}]	$\hat{\mu}_{MSD}$	$\hat{\mu}_{\tau_n}$	$\hat{\mu}_{\hat{\tau}_n}$	$\hat{\mu}_{\hat{\ell}_n}$	$\hat{\mu}_{C_v}$
1.00	5.03 ± 0.97	1.0509 ± 0.0002	0.93 ± 0.09	0.94 ± 0.08	0.95 ± 0.06	1.039 ± 0.010
1.25	4.88 ± 0.95	1.2578 ± 0.0003	1.30 ± 0.05	1.29 ± 0.07	1.22 ± 0.07	1.200 ± 0.013
1.50	5.03 ± 0.74	1.5473 ± 0.0004	1.49 ± 0.04	1.46 ± 0.06	1.45 ± 0.05	1.434 ± 0.005
1.75	5.11 ± 0.75	1.7498 ± 0.0003	1.71 ± 0.06	1.70 ± 0.06	1.71 ± 0.09	1.704 ± 0.001
2.00	4.46 ± 0.93	1.9158 ± 0.0003	2.05 ± 0.06	2.00 ± 0.08	2.03 ± 0.07	1.964 ± 0.001

Table S3: Anomalous diffusion exponent of fractional Brownian walks. Estimated anomalous diffusion exponent $\hat{\mu}$ for fractional Brownian walks (Figure 4) based on fitting the mean squared displacement ($\hat{\mu}_{MSD}$) and the velocity autocorrelation function ($\hat{\mu}_{C_v}$). The first column shows the corresponding ground truth value of μ. Corresponding instantaneous microrobot's speed $\langle \hat{v} \rangle$ are also shown. Errors represent one standard deviation of the respective fit parameters.

μ	$\langle \hat{v} \rangle$ [µm s^{-1}]	$\hat{\mu}_{MSD}$	$\hat{\mu}_{C_v}$
0.33	3.92 ± 0.76	0.36944 ± 0.00030	0.29 ± 0.08
0.66	4.99 ± 0.84	0.67492 ± 0.00028	0.67 ± 0.09
1.00	5.66 ± 0.82	0.95485 ± 0.00022	1.00 ± 0.08
1.33	4.98 ± 0.78	1.29699 ± 0.00023	1.27 ± 0.06
1.66	4.60 ± 0.91	1.67541 ± 0.00021	1.60 ± 0.05
1.99	3.71 ± 0.56	1.98648 ± 0.00003	2.00 ± 0.06

Supporting Videos

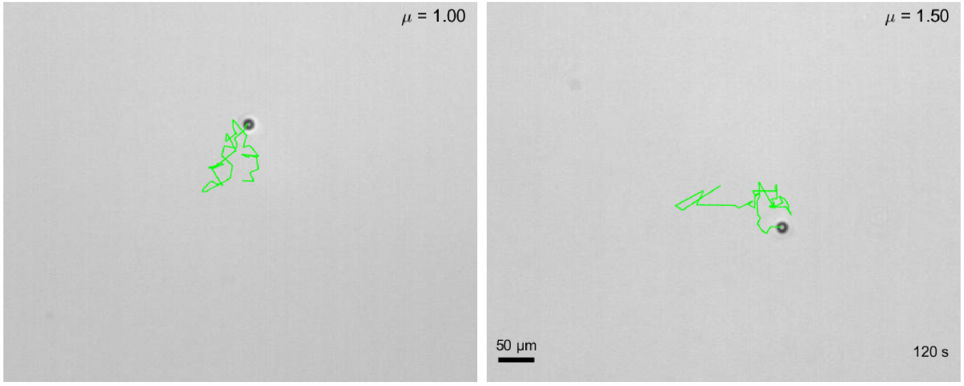

Movie 1: Normal diffusion vs Lévy walk. Exemplary 120-s traces of a microrobot performing normal diffusion (anomalous diffusion exponent $\mu = 1$) and one performing a Lévy walk with $\mu = 1.5$. The second trace shows that the microrobot performs occasional long jumps displaying the typical spatial features of Lévy walks.

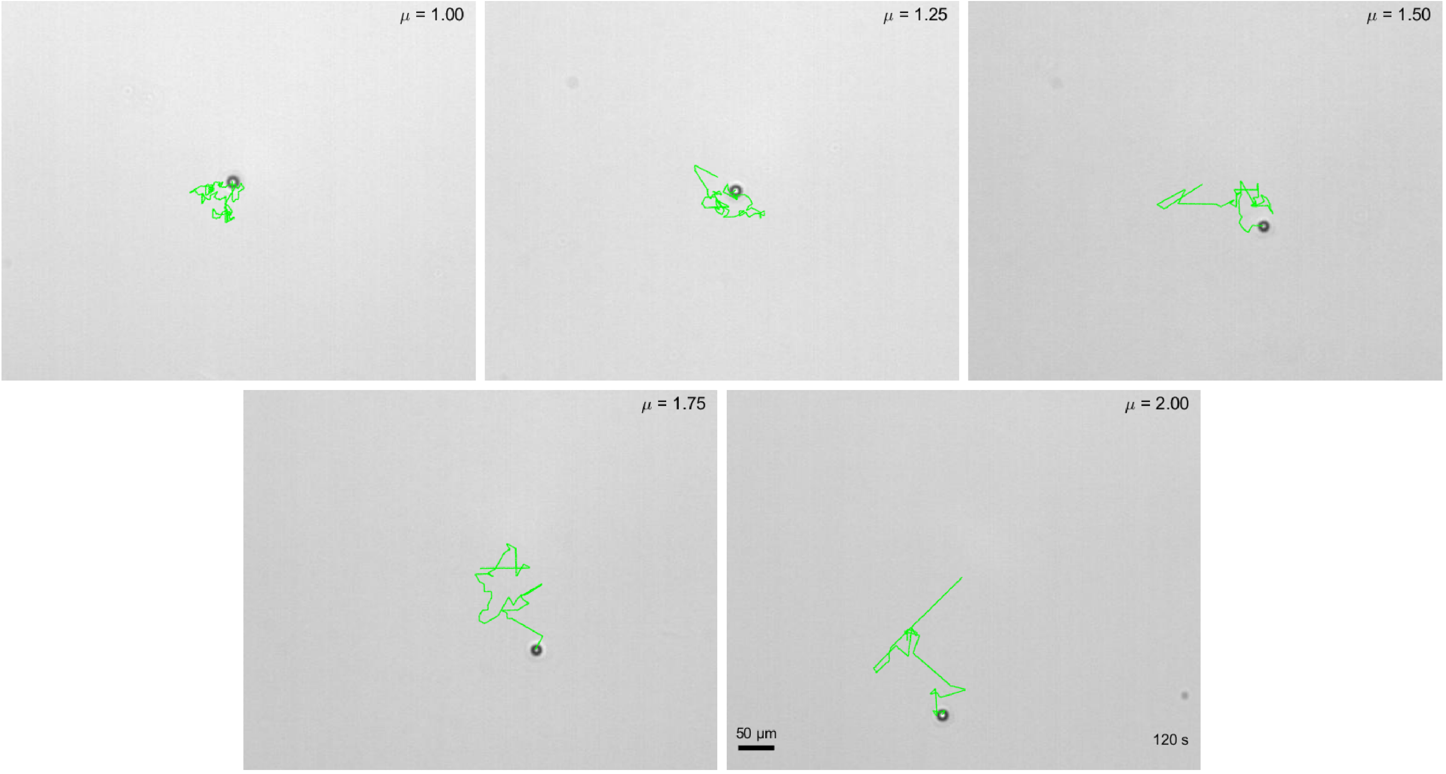

Movie 2: Lévy walks. Exemplary 120-s traces of microrobots performing Lévy walks with different anomalous diffusion exponent μ. As μ increases from the diffusive ($\mu = 1$) to the ballistic limit ($\mu = 2$), the microrobot tends to move ballistically over longer distances before a random change in orientation occurs.

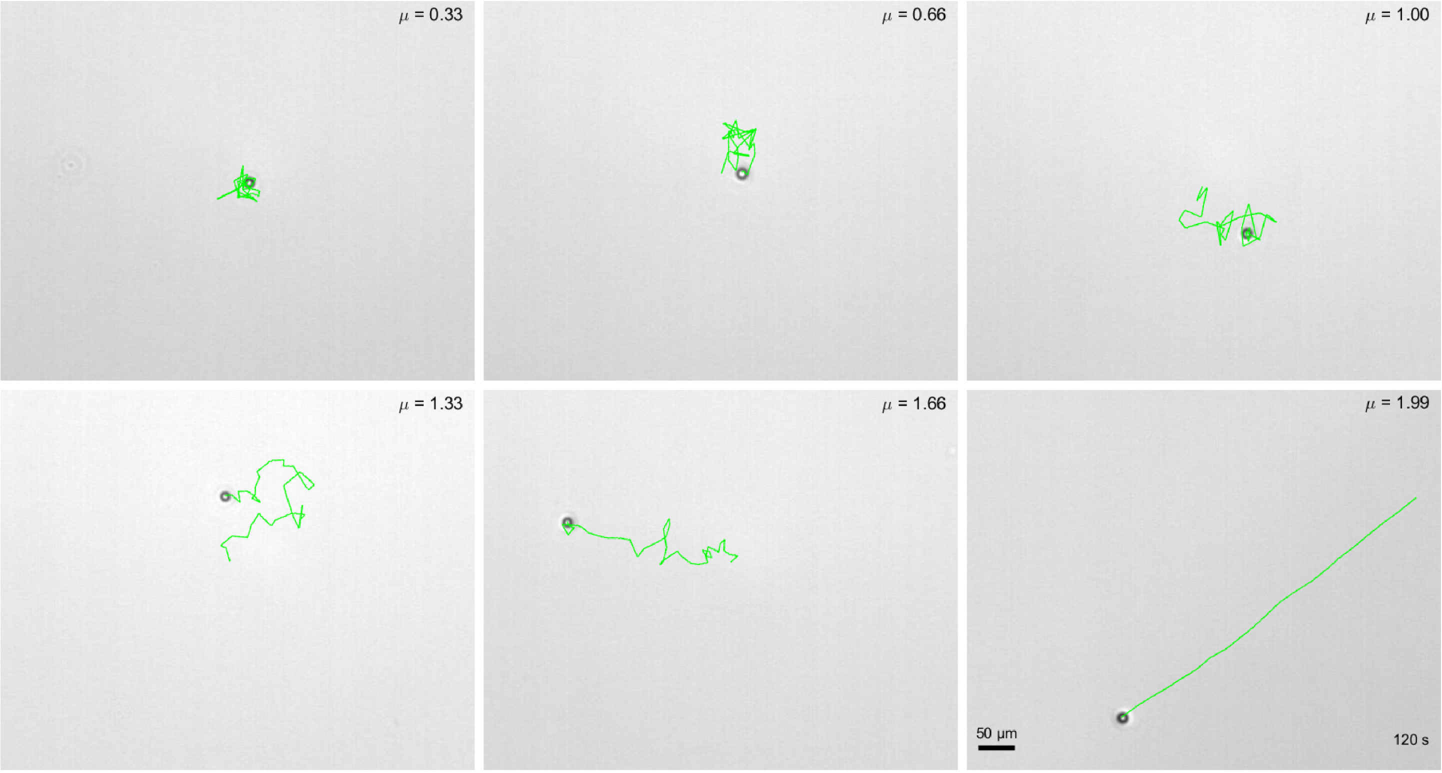

Movie 3: Fractional Brownian walks. Exemplary 120-s traces of microrobots performing fractional Brownian walks with different anomalous diffusion exponent μ. As μ increases from the subdiffusive ($\mu = 0.33$) to the ballistic limit ($\mu = 2$), the microrobot's motion becomes less and less localized: the microrobot's tendency to turn backward (negative persistence) reduces in favor of its forward propagation (positive persistence) (Figure S9).

Supporting References

[72] P. Romanczuk, M. Bär, W. Ebeling, B. Lindner, L. Schimansky-Geier, Active brownian particles, *Eur. Phys. J. Spec. Top.* **2012**, *202* 1.

[73] V. Cranna, Karl Pearson and sir Ronald Ross, **2015**, URL `https://blogs.lshtm.ac.uk/library/2015/03/27/karl-pearson-and-sir-ronald-ross/`, Library, Archive and Open Research Services Blog.

[74] K. Pearson, The problem of the random walk, *Nature* **1905**, *72* 294, 342.

[75] K. Pearson, Mathematical contributions to the theory of evolution - a mathematical theory of random migration, *Biometric Ser.* **1906**, *3* 54.

[76] C. Gardiner, *Stochastic Methods: A Handbook for the Natural and Social Sciences*, Springer Series in Synergetics. Springer, Berlin, **2009**.

[77] F. Biagini, Y. Hu, B. Øksendal, T. Zhang, *Stochastic Calculus for Fractional Brownian Motion and Applications*, Probability and Its Applications. Springer, London, **2008**.

[78] P. Embrechts, *Selfsimilar processes*, Princeton University Press, Princeton, New Jersey, USA, **2009**.

[79] F. Molz, H. Liu, Fractional Brownian motion and fractional Gaussian noise in subsurface hydrology: A review, presentation of fundamental properties, and extensions, *Water Res. Res.* **1997**, *33*, 10 2273.
